# Enoxaparin augments alpha-1-antitrypsin inhibition of TMPRSS2, a promising drug combination against COVID-19

**DOI:** 10.1038/s41598-022-09133-9

**Published:** 2022-03-25

**Authors:** Xiyuan Bai, Ashley M. Buckle, Eszter K. Vladar, Edward N. Janoff, Reeti Khare, Diane Ordway, David Beckham, Lorelenn B. Fornis, Abraham Majluf-Cruz, Randolph V. Fugit, Brian M. Freed, Soohyun Kim, Robert A. Sandhaus, Edward D. Chan

**Affiliations:** 1 Department of Medicine, Rocky Mountain Regional Veterans Affairs Medical Center, Aurora, CO USA; 2 Department of Pharmacy, Rocky Mountain Regional Veterans Affairs Medical Center, Aurora, CO USA; 3 Department of Academic Affairs and Medicine, National Jewish Health, Denver, CO USA; 4 Mycobacteriology Laboratory, Advance Diagnostics, National Jewish Health, Denver, CO USA; 5 Division of Pulmonary Sciences and Critical Care Medicine, University of Colorado Anschutz Medical Campus, Aurora, CO USA; 6 Division of Infectious Diseases, University of Colorado Anschutz Medical Campus, Aurora, CO USA; 7 Department of Immunology, University of Colorado Anschutz Medical Campus, Aurora, CO USA; 8 Department of Microbiology, Immunlogy, and Pathology, Colorado State University, Fort Collins, CO USA; 9 Department of Biochemistry and Molecular Biology, Biomedicine Discovery Institute, Monash University, Clayton, VIC Australia; 10 Laboratory of Cytokine Immunology, Department of Biomedical Science and Technology, Konkuk University, Seoul, South Korea; 11 College of Veterinary Medicine, Konkuk University, Seoul, South Korea; 12 Unidad de Investigacion Medica en Trombosis, Hemostasia y Aterogenesis, Instituto Mexicano del Seguro Social, Mexico City, Mexico; 13 National Jewish Health, D509, Neustadt Building, 1400 Jackson Street, Denver, CO 80206 USA

**Keywords:** Viral infection, Molecular modelling

## Abstract

The cell surface serine protease Transmembrane Protease 2 (TMPRSS2) is required to cleave the spike protein of SARS-CoV-2 for viral entry into cells. We determined whether negatively-charged heparin enhanced TMPRSS2 inhibition by alpha-1-antitrypsin (AAT). TMPRSS2 activity was determined in HEK293T cells overexpressing TMPRSS2. We quantified infection of primary human airway epithelial cells (hAEc) with human coronavirus 229E (HCoV-229E) by immunostaining for the nucleocapsid protein and by the plaque assay. Detailed molecular modeling was undertaken with the heparin–TMPRSS2–AAT ternary complex. Enoxaparin enhanced AAT inhibition of both TMPRSS2 activity and infection of hAEc with HCoV-229E. Underlying these findings, detailed molecular modeling revealed that: *(i)* the reactive center loop of AAT adopts an inhibitory-competent conformation compared with the crystal structure of TMPRSS2 bound to an exogenous (nafamostat) or endogenous (HAI-2) TMPRSS2 inhibitor and *(ii)* negatively-charged heparin bridges adjacent electropositive patches at the TMPRSS2–AAT interface, neutralizing otherwise repulsive forces. In conclusion, enoxaparin enhances AAT inhibition of both TMPRSS2 and coronavirus infection. Such host-directed therapy is less likely to be affected by SARS-CoV-2 mutations. Furthermore, given the known anti-inflammatory activities of both AAT and heparin, this form of treatment may target both the virus and the excessive inflammatory consequences of severe COVID-19.

## Introduction

Despite the availability of efficacious COVID-19 vaccines, as of January 2022 the number of COVID-19 cases and hospitalizations are rising in the U.S. and other parts of the world. Thus, effective treatment for severe COVID-19 is a top priority but remains elusive. Among the array of agents being studied against COVID-19, alpha-1-antitrypsin (AAT)—a member of a superfamily of serine protease inhibitors (serpin) and the most abundant serpin in circulation^1^—has been shown to inhibit Transmembrane Protease, Serine 2 (TMPRSS2), a cell surface serine protease that is required to process the spike protein of SARS-CoV-2 to allow the virus to gain intracellular entry^2–8^.

Negatively-charged polysaccharides augment serpin activity by introducing a more favorable electrostatic interaction between the serpin and its protease, forming a ternary complex of polysaccharide–protease–serpin. For example, the negatively-charged polysaccharide dextran binds to multiple positively-charged amino acids on the F1-helix of the serpin C1-esterase (C1s) inhibitor (C1INH), as well as to positively-charged amino acids of the autolysis loop of C1s serine protease, facilitating the binding of C1INH to C1s as well as to another serine protease kallikrein^9^. As a result, ternary complexes of dextran–C1s–C1INH or dextran–kallikrein–C1INH are formed, resulting in enhanced serpin activity for its cognate protease. The activity of C1INH on C1s is also augmented by heparin, a polysaccharide with the highest negative charge density of any known biological molecules^10^.

Epidemiologic studies indirectly support the paradigm that AAT antagonizes SARS-CoV-2 infection. COVID-19 cases are increased in areas of Italy with an increased prevalence of AAT deficiency^11^. AAT deficient subjects were 8.8-fold more likely to have symptomatic COVID-19 than the general Italian population^12^. Shapira and colleagues^13^ found a significant direct correlation between the frequency of the protease inhibitor (Pi)Z and PiS alleles with COVID-19 death rates in 67 countries. Yoshikura^14^ reported a robust correlation between the Pi*Z variant and the number of COVID-19 cases (correlation coefficient (CC) = 0.8584) and deaths (CC = 0.8713) in 68 countries. McElvaney and co-workers^15^ found that the interleukin-6 (IL-6):AAT ratio is markedly elevated in critically ill patients with COVID-19 compared with healthy volunteers or stable hospitalized COVID-19 patients; this ratio also directly correlated with prolonged hospital stay and mortality.

In light of the aforementioned polysaccharide–serine protease–serpin paradigm and epidemiologic evidence, we investigated whether unfractionated heparin (UFH), enoxaparin, and nadroparin—the latter two being low molecular fractions of UFH—augment AAT inhibition of TMPRSS2. We found that enoxaparin significantly increased AAT inhibition of both TMPRSS2 and a human coronavirus infection of human airway epithelial cells. These biochemical and biological synergies are strongly supported by state-of-the-art molecular modeling wherein highly negatively-charged heparin bridges adjacent electropositive patches at the TMPRSS2–AAT interface, enhancing their interaction.

## Results

### Overexpression of TMPRSS2 in HEK293T cells increases its serine protease activity

**Figure 1:**
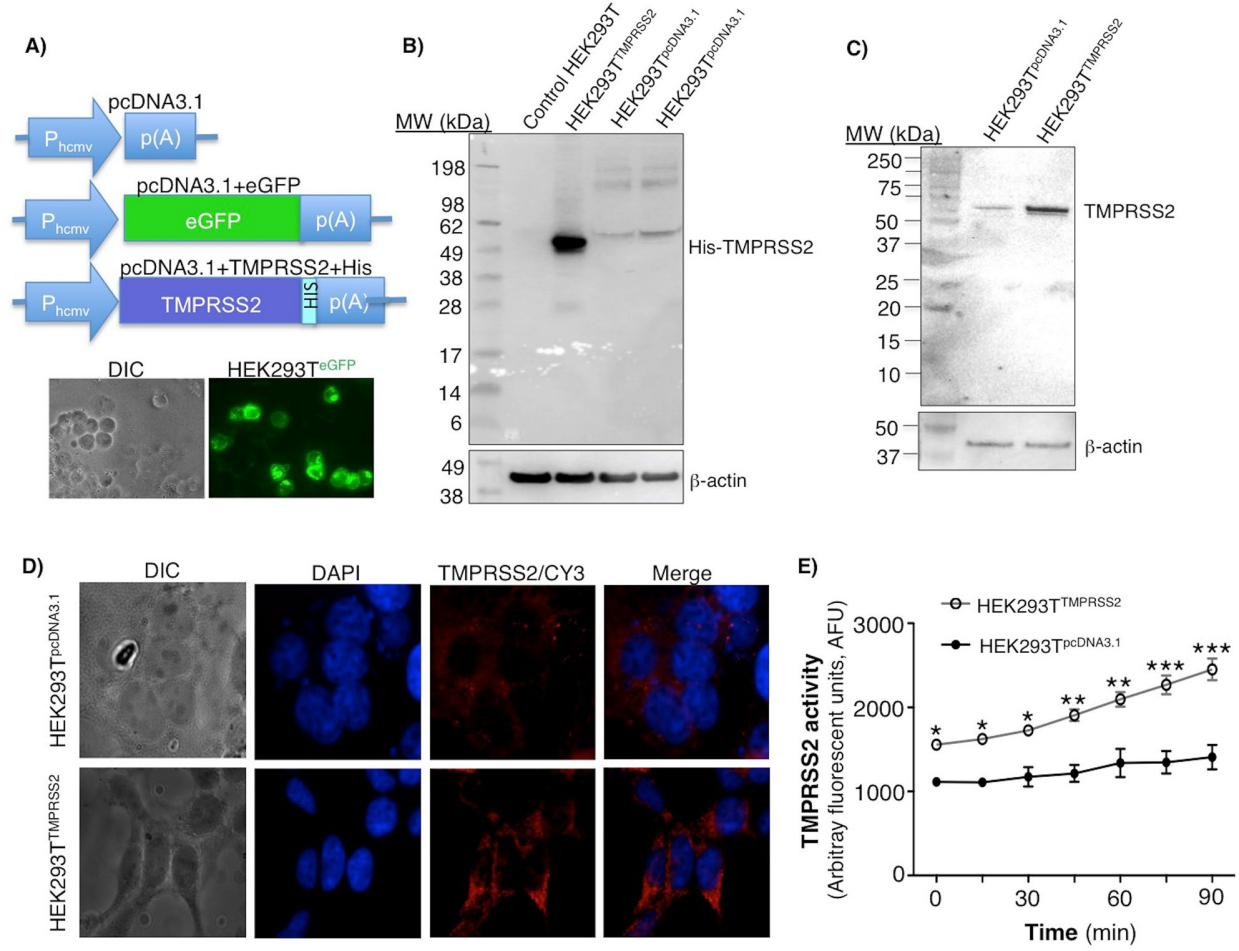
Overexpression of recombinant TMPRSS2 in HEK293T cells. (**A**) pcDNA3.1 empty vector, pcDNA3.1^eGFP^, or pcDNA3.1^TMPRSS2+His^ plasmids (top panel) used to transfect HEK293T cells. Photomicrograph of pcDNA3.1^eGFP^ transfected cells (HEK293T^eGFP^) (bottom panel). Cell lysates were prepared and immunoblotted with (**B**) anti-His antibody or (**C**) anti-TMPRSS2 antibody. (**D**) Immunocytochemistry staining to detect TMPRSS2 in control (HEK293T^pcDNA3.1^) and TMPRSS2–transfected HEK293T (HEK293T^TMPRSS2^) cells. (**E**) Spontaneous TMPRSS2 activity using the fluorogenic substrate Boc-QAR-AMC as a function of time in the HEK293T^pcDNA3.1^ and HEK293T^TMPRSS2^ cells. Data shown are representative or triplicate means ± SEM of three independent experiments. *p < 0.05, **p < 0.01, ***p < 0.001 compared to HEK293T^pcDNA3.1^ cells.

HEK293T cells were transfected with pcDNA3.1 empty vector (HEK293T^pcDNA3.1^), pcDNA3.1^eGFP^ plasmid (HEK293T^eGFP^), or pcDNA3.1^TMPRSS2+His^ plasmid (HEK293T^TMPRSS2^). Transfection efficiency was robust as evinced by the abundant fluorescence of HEK293T^eGFP^ cells (Fig. 1A, bottom panel). Compared to control HEK293T^pcDNA3.1^ cells, HEK293T^TMPRSS2^ cells strongly expressed TMPRSS2 as shown by immunoblot with anti-His-(TMPRSS2) and anti-TMPRSS2 antibodies and by immunocytofluorescent analysis for TMPRSS2 (Fig. 1B–D). To analyze TMPRSS2 activity, we incubated the cells with the TMPRSS2 substrate Boc-QAR-AMC and quantified the amount of fluorogenic catalytic product liberated. HEK293T^TMPRSS2^ cells had significantly greater TMPRSS2 activity than HEK293T^pcDNA3.1^ cells (Fig. 1E).

### AAT inhibits TMPRSS2 activity in a dose-dependent fashion

**Figure 2:**
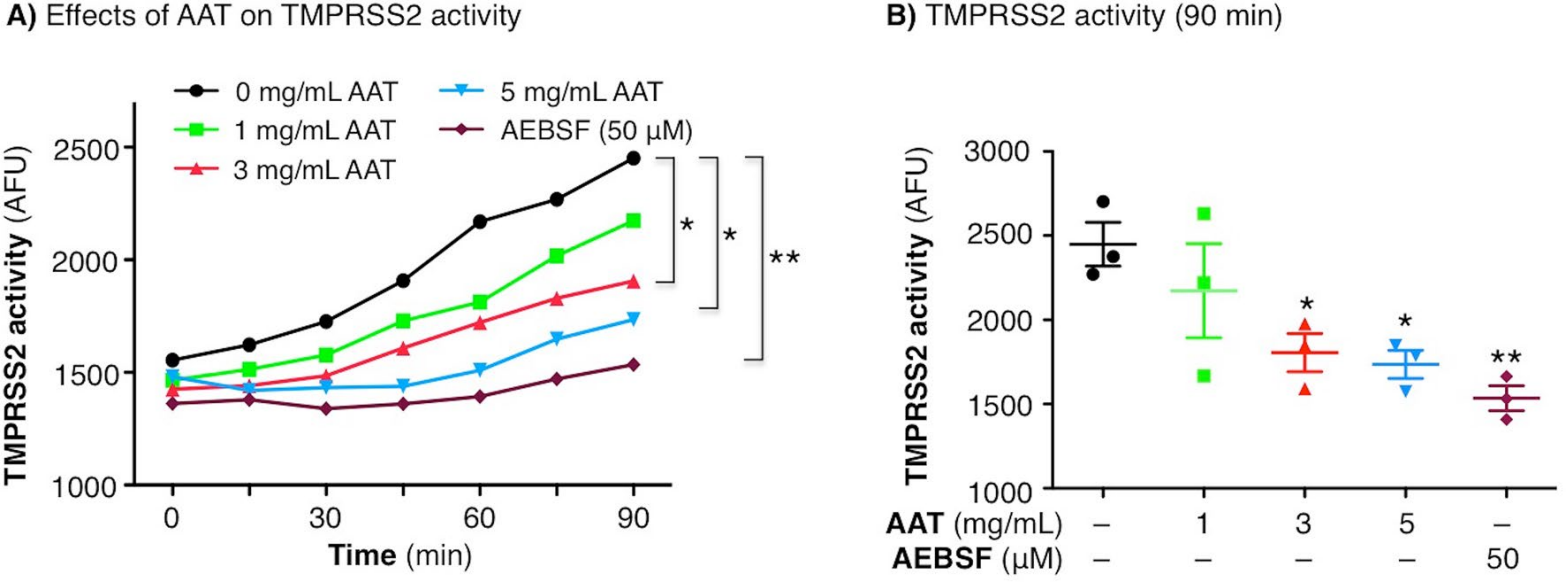
Activity of TMPRSS2 in the presence of AAT. (**A**) HEK293T^TMPRSS2^ cells were left untreated or pre-treated with AAT at the indicated final concentrations or AEBSF (50 µM) for 30 min, followed by addition of the fluorogenic substrate Boc-QAR-AMC. Fluorescence was measured immediately on the fluorescent plate reader and then every 15 min for a total of 90 min. (**B**) TMPRSS2 activity at 90 min. Data shown are the triplicate means ± SEM of three independent experiments. *p < 0.05, **p < 0.01 at 90 min compared to untreated cells. AAT = alpha-1-antitrypsin.

TMPRSS2 activity was then compared between untreated HEK293T^TMPRSS2^ cells with those incubated for 30 min with 1, 3, or 5 mg/mL of AAT (19.2, 57.7, and 96.2 µM, respectively), physiologic concentrations found in plasma. Compared with untreated cells, AAT reduced TMPRSS2 activity in a concentration-dependent fashion (Fig. 2A) with the 90 min time point highlighted separately (Fig. 2B). AEBSF, a potent serine protease inhibitor, strongly inhibited TMPRSS2 activity (Fig. 2B).

### Enoxaparin enhances AAT inhibition of TMPRSS2 activity

**Figure 3:**
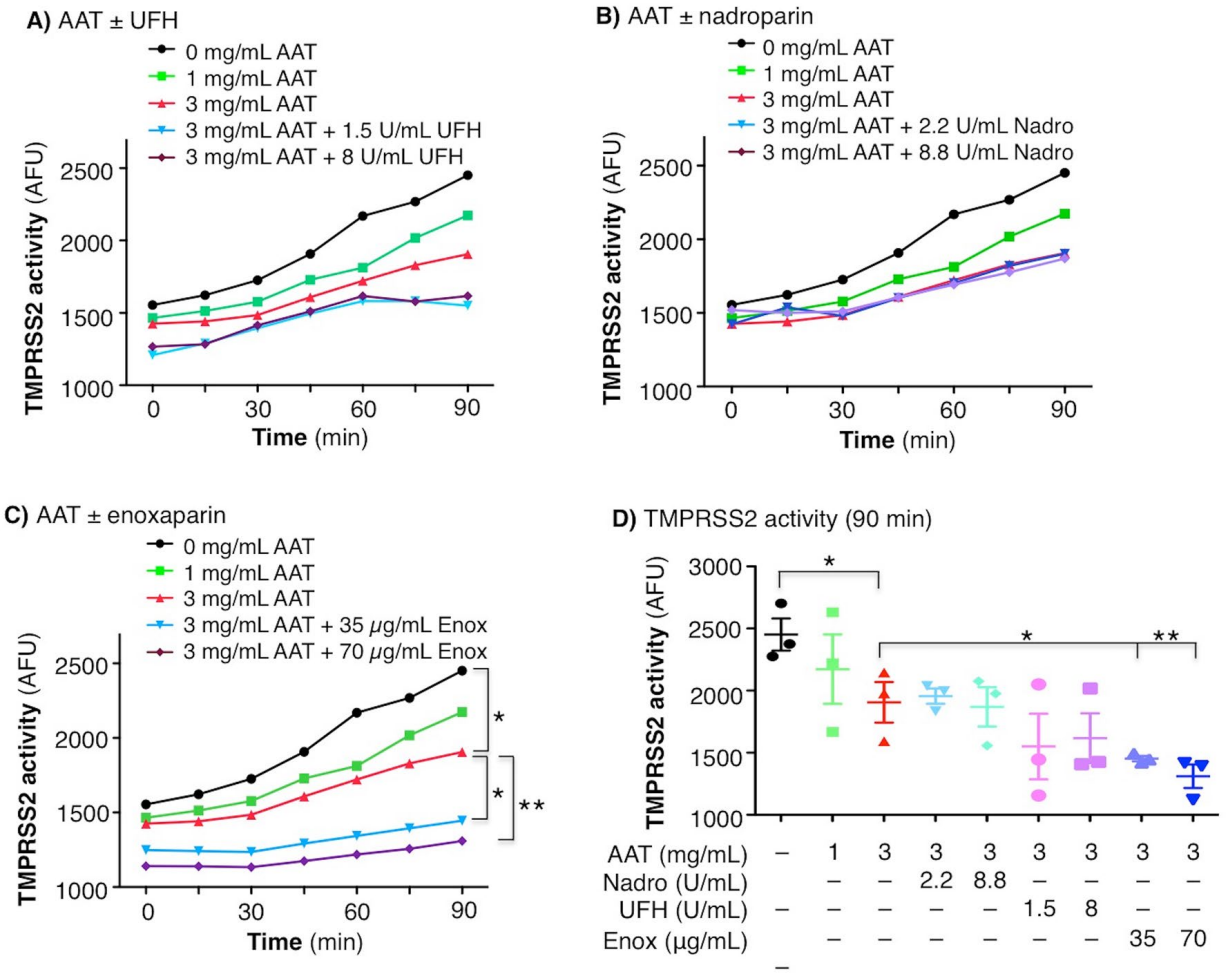
Activity of TMPRSS2 in the presence of AAT alone or with UFH, nadroparin, or enoxaparin. HEK293T^TMPRSS2^ cells were pre-treated with AAT alone at the indicated concentrations or with (**A**) UFH, (**B**) nadroparin (Nadro), or (**C**) enoxaparin (Enox) at the indicated final concentrations for 30 min, followed by addition of the fluorescent substrate Boc-QAR-AMC. Fluorescence was measured immediately on the fluorescent plate reader and then every 15 min for a total of 90 min. (**D**) TMPRSS2 activity at 90 min in untreated cells and cells incubated with AAT ± two concentrations of nadroparin, UFH, and enoxaparin. Data shown are the triplicate means ± SEM of three independent experiments. *p < 0.05, **p < 0.01 at 90 min. Unfractionated heparin = UFH.

To determine the effects of UFH, enoxaparin, or nadroparin, the cells were left untreated or pre-incubated for 30 min with AAT alone at 1 and 3 mg/mL, or with combined 3 mg/mL AAT with two different concentrations of UFH, nadroparin, or enoxaparin. UFH at both concentrations elicited a modest further reduction of TMPRSS2 activity compared with AAT 3 mg/mL alone, particularly at the longer incubation times (Fig. 3A), whereas nadroparin did not (Fig. 3B). In contrast, enoxaparin + AAT most potently inhibited TMPRSS2 activity in a dose-dependent manner compared with AAT alone (Fig. 3C,D). Because the HEK293T^TMPRSS2^ cells were pre-treated with both AAT and enoxaparin for 30 min, it is not unexpected that there would be decreased TMPRSS2 activity from the onset of the assay.

**Figure 4:**
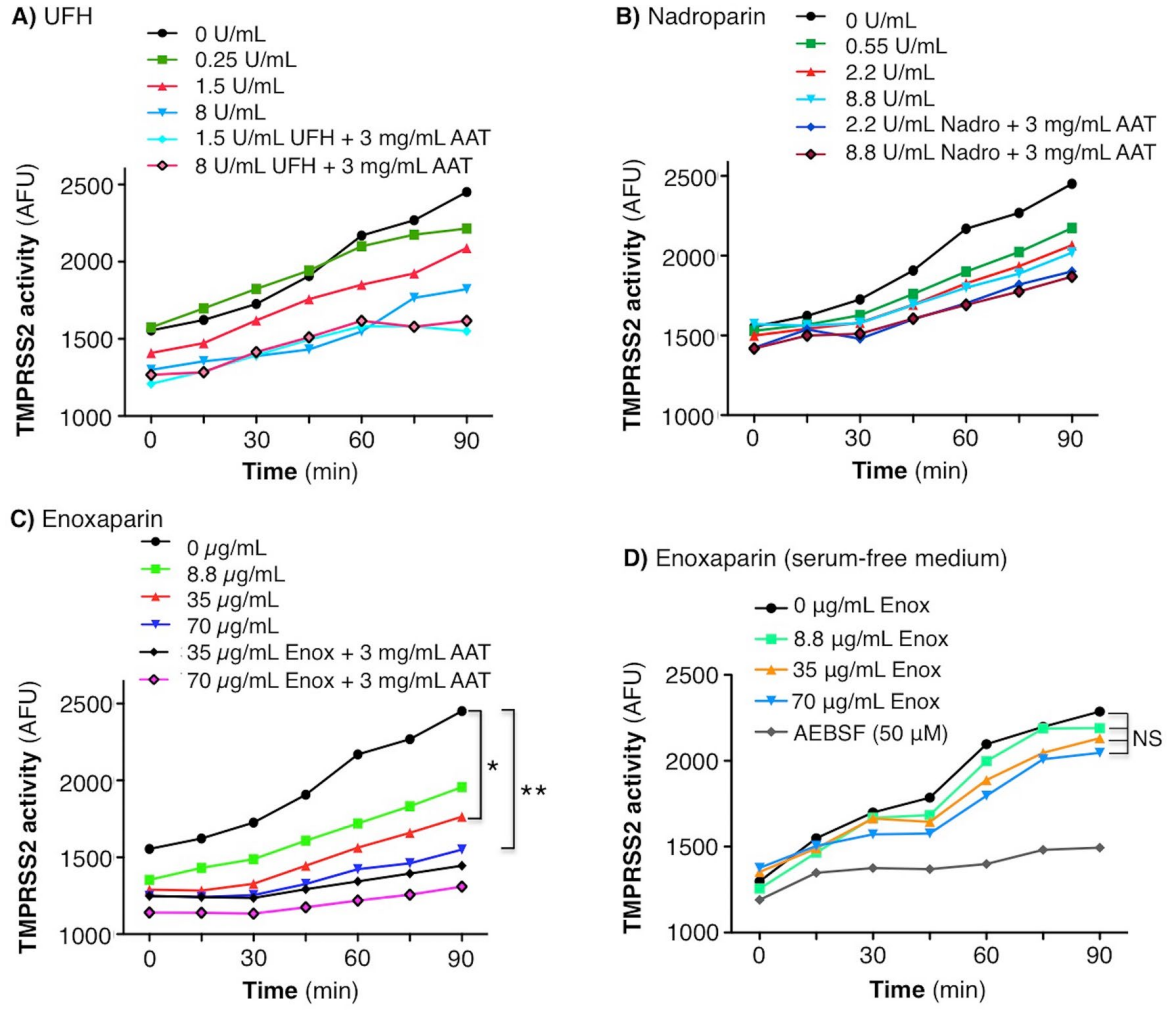
Activity of TMPRSS2 in the presence of UFH, nadroparin, or enoxaparin alone or with AAT. HEK293T^TMPRSS2^ cells were pre-treated with (**A**) UFH, (**B**) nadroparin, or (**C**) enoxaparin ± AAT at the indicated final concentrations for 30 min, followed by addition of the fluorescent substrate Boc-QAR-AMC. (**D**) HEK293T^TMPRSS2^ cells were grown in Gibco 293 Serum-free Medium II, pre-treated with enoxaparin at the indicated concentrations for 30 min, followed by addition of Boc-QAR-AMC. Fluorescence was measured immediately on the fluorescent plate reader and then every 15 min for a total of 90 min. Data shown are the mean ± SEM of three independent experiments. *p < 0.05, **p < 0.01 at 90 min.

We next determined whether the heparins alone also inhibited TMPRSS2. Unexpectedly, we found that UFH alone had a modest dose–response inhibitory effect on TMPRSS2 activity (Fig. 4A). Nadroparin had minimal impact and only at higher nadroparin concentrations at the longer time points (Fig. 4B). Enoxaparin alone at 35 and 70 µg/mL significantly inhibited TMPRSS2 activity at the 90 min incubation period (Fig. 4C). Because the experiments were performed in cell culture medium that includes fetal bovine serum that is likely to contain native AAT (and perhaps other serpins), we performed the experiments with enoxaparin alone in Gibco 293 Serum-free Medium II. In the absence of serum, enoxaparin had little or no inhibitory effect on TMPRSS2 activity (Fig. 4D).

As predicted, AAT was found to be a very potent inhibitor of elastase activity (Figs. S1, S2). Moreover, there was no further inhibition of elastase activity by AAT with addition of any of the heparins.

### Enoxaparin augments AAT inhibition of HCoV-229E infection of primary human airway epithelial cells

**Figure 5:**
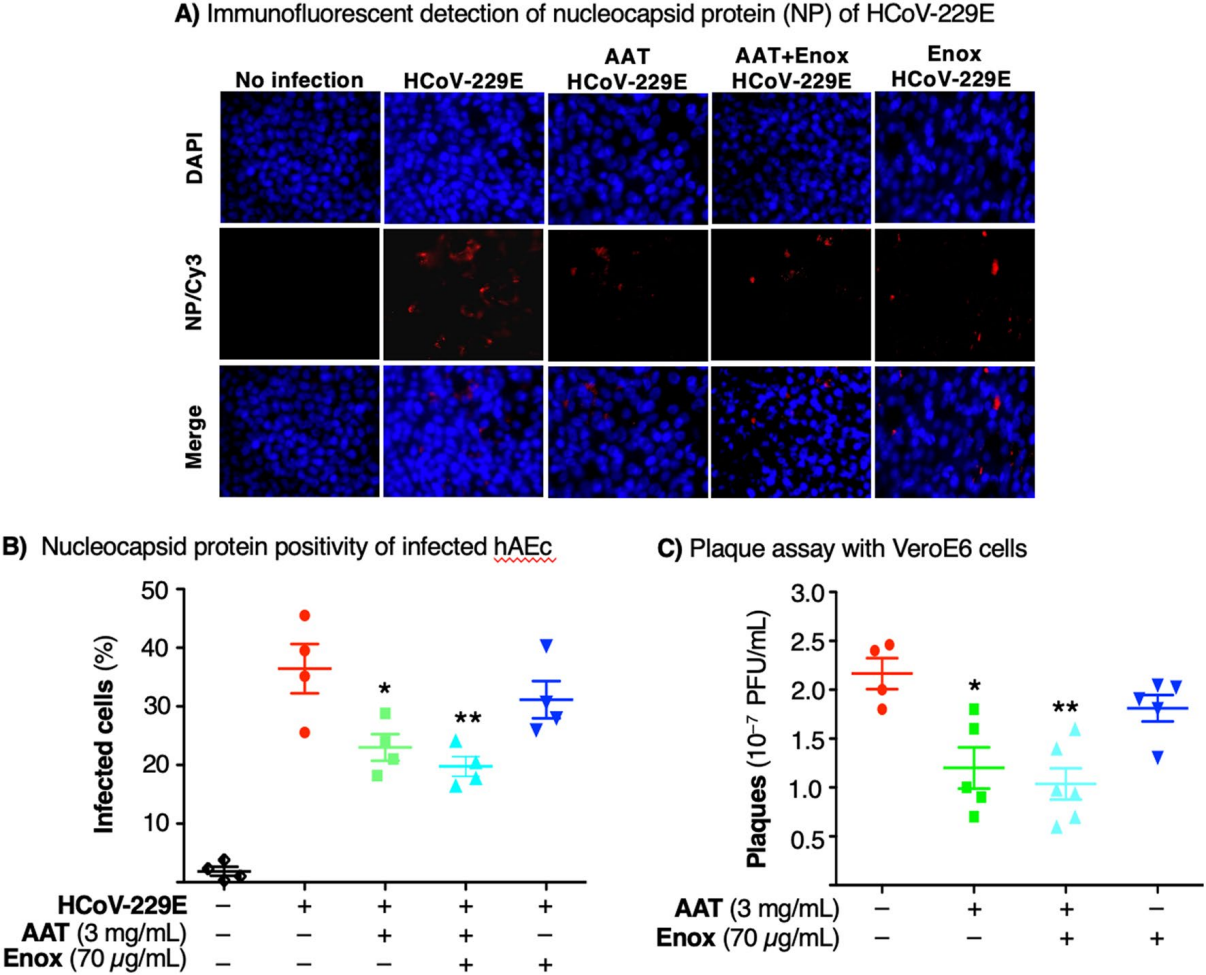
Effects of AAT, enoxaparin, or both on infection of hAEc with HCoV-229E. (**A**) Immunofluorescence analysis of HCoV-229E-infected hAEc grown in air–liquid interface. The hAEc were pre-treated with AAT (3 mg/mL), enoxaparin (70 µg/mL), or both for 1 h, and then infected with HCoV-229E at a multiplicity-of-infection of 1 hAEc:0.01 HCoV-229E. Three days after infection, the cells were fluorescently immunostained for the nucleocapsid protein of HCoV-229E. The nuclei were stained with DAPI. Fluorescent images were taken at a magnification of 400X by confocal microscopy (Carl Zeiss Anxiovert 200 M). (**B**) Percentage of HCoV-229E-infected hAEc with > 700 total cells counted for each condition. Data shown are the mean ± SEM of three independent experiments. *p < 0.05, **p < 0.01 compared to HCoV-229E infection alone. (**C**) Plaque assay. The apical chamber medium of HCoV-229E-infected hAEc were used to infect VeroE6 cells and incubated for 4–5 days. Infection of the hAEc were done in triplicates and subsequent infection of VeroE6 cells with the supernatant of HCoV-229E-infected hAEc were each done in triplicates. Thus, the data shown are the triplicate means ± SEM of the original triplicate experiments. *p < 0.05 and **p < 0.01 compared to cells infected with HCoV-229E only without treatment. hAEc = primary human airway epithelial cells, HCoV-229E = human coronavirus 229E.

To determine the effects of AAT, enoxaparin, or both on coronavirus infection, we quantified viral load by immunofluorescent staining for the nucleocapsid protein and by the plaque assay of primary human airway epithelial cells (hAEc) cultured in air–liquid interface infected with the human coronavirus 229E (HCoV-229E). Compared with no infection, cells infected with HCoV-229E immunostained positively for the viral nucleoprotein (Fig. 5A,B). Pre-treatment of hAEc with AAT, enoxaparin, or both, reduced the number of cells that stained positive for the nucleoprotein.

The burden of HCoV-229E was also quantified by the plaque assay in which the supernatant from the apical chambers of the uninfected and infected hAEc in air–liquid interface were used to infect VeroE6 cells. Incubation of the VeroE6 cells with 10^–7^ dilution of the supernatant of HCoV-229E-infected hAEc demonstrated visually quantifiable plaques (Fig. 5C). In VeroE6 cells incubated with medium from HCoV-229E-infected hAEc treated with AAT, there was a modest but significant reduction in the number of plaques and a further decrease with both AAT and enoxaparin. Interestingly, there was also a modest but consistent decrease in the number of plaques with enoxaparin alone.

### Structural rationalization of heparin augmenting AAT inhibition of TMPRSS2

**Figure 6:**
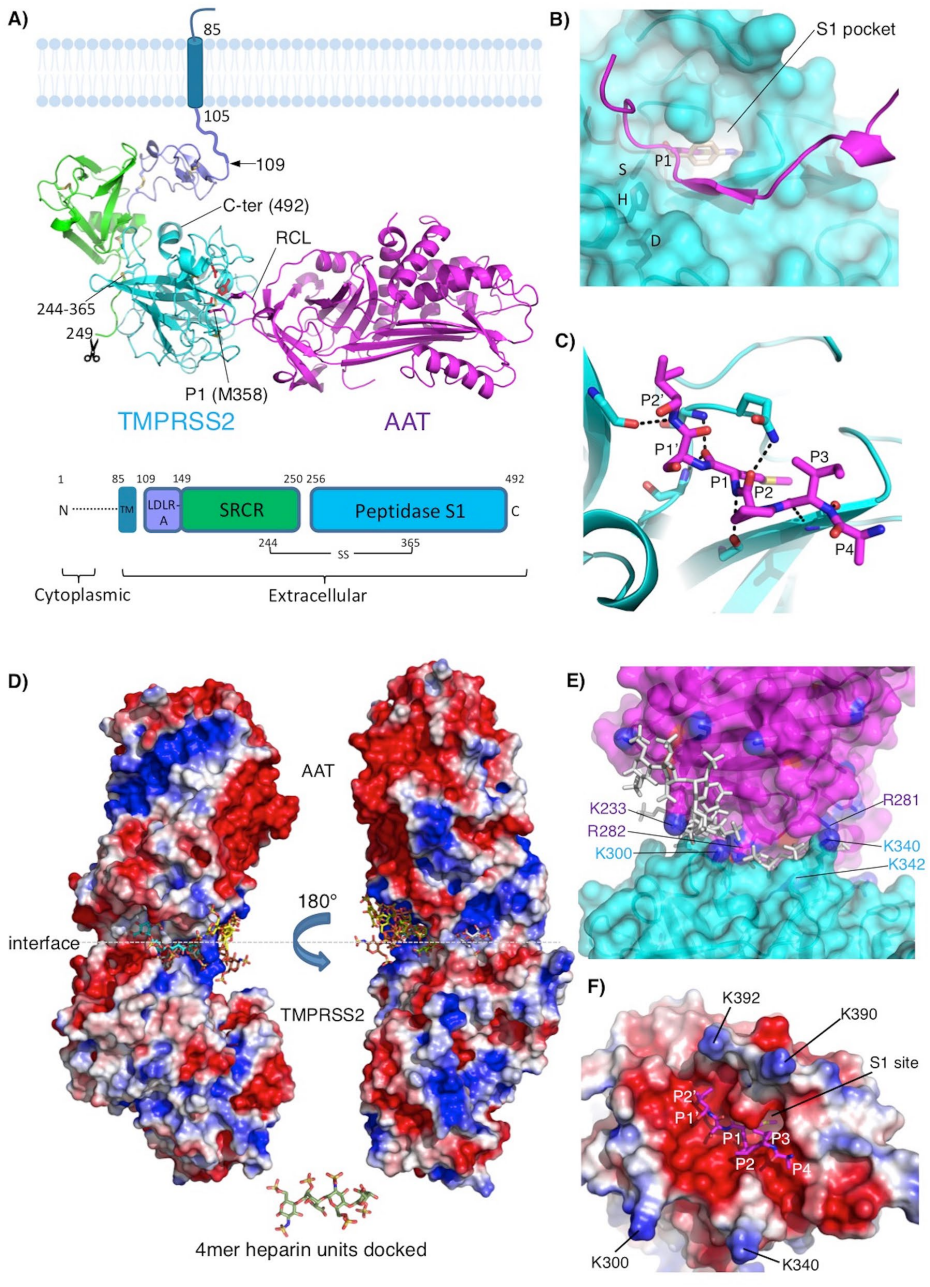
Modeling TMPRSS2 and its interaction with AAT and heparin. (**A**) Model of the TMPRSS2–AAT Michaelis complex. TMPRSS2 domain organization is shown in inset (TM = transmembrane region; residue numbers of domain boundaries are indicated). Domains are colored separately: low-density lipoprotein receptor A (LDLR-A, plum), scavenger receptor cysteine-rich repeat (SRCR, green), peptidase S1 (catalytic) domain (cyan). Disulphides are shown as sticks. A conserved disulphide formed between the catalytic domain and SRCR domain (244–365) is indicated. Scissors indicate the cleavage site at R255 at the N-terminus of the catalytic domain that generates the mature form. N- and C-termini are labeled. The catalytic triad (H296, D345 and S441) are shown as red sticks. TM region in lipid bilayer is also indicated as a schematic, indicating proximity to the plasma membrane, resembling the homologue hepsin. Reactive centre loop (RCL of AAT, bound to the active site of TMPRSS2 is labeled. (**B**) Molecular surface of TMPRSS2 showing bound RCL (magenta cartoon). Catalytic residues are labelled. P1 sidechain of AAT occupies the S1 site of TMPRSS2, superimposed on the TMPRSS2 inhibitor nafamostat (wheat). (**C**) Hydrogen-bonding interactions between the RCL of AAT and TMPRSS2. Black broken lines indicate hydrogen bonds. (**D**) Heparin molecules stabilize TMPRSS2–AAT association by acting as electrostatic bridges. Electrostatic potential surfaces of the TMPRSS2–AAT complex (blue = positive, red = negative) showing docked heparin 4mers binding to electropositive patches on the molecular surface. Carbon atoms of each 4mer heparin are coloured differently. (**E**) Location of Lys/Arg residues (sticks: AAT = magenta; TMPRSS2 = cyan) in the vicinity of heparin (grey sticks) binding sites at the TMPRSS2(cyan)–AAT(magenta) interface. Semi-transparent molecular surface is shown, and Lys/Arg residues that contribute to unfavorable electrostatics at the interface are shown and labelled (sidechain nitrogen atoms colored blue). (**F**) Poor electrostatic complementarity at P2′, P1′, P1, P2, P3, and P4 of the RCL (magenta) in the TMPRSS2 active site. P1 residue of the AAT RCL and cognate S1 pocket of TMPRSS2 are labeled. View is orthogonal to (**E**) showing the lysines located at the interface rim that are unfavorably close to positively charged residues of AAT.

To investigate the structural basis for the observed effect of heparin on AAT inhibition of TMPRSS2, we performed a rigorous modeling analysis of the participating molecules. First, we created a near-complete model of the extracellular region of TMPRSS2, which consists of three domains: low-density lipoprotein receptor A (LDLR-A) domain, scavenger receptor cysteine-rich repeat (SRCR) domain, and a C-terminal peptidase S1 (catalytic) domain (Fig. 6A). The full-length protein is displayed on the cell surface, with cleavage at the N-terminus of the catalytic domain (R255)^16^, generating two chains that are held together by a conserved disulfide bond, such that the molecule remains membrane-bound after activation^17^ (Fig. 6A), resembling the homologue hepsin^18^. The serine protease domain contains the catalytic triad H296, D345, and S441 and adopts the conserved fold of the trypsin-like (S1 family) of serine proteases.

We next created a model of TMPRSS2 in complex with AAT (Figs. 6 and S3). Excellent alignment between the P1 residue of the reactive center loop (RCL) of AAT and the TMPRSS2 inhibitor nafamostat indicates that AAT is docked with TMPRSS2 in an inhibition-competent orientation (Fig. 6B,C). Nafamostat is a TMPRSS2 inhibitor under investigation as a potential COVID-19 therapeutic^19^. Notably, the methionine sidechain at P1 is bound in the S1 site that is occupied by the phenylguanidino moiety of nafamostat in the TMPRSS2 crystal structure^20^ (Fig. 6B). Electrostatic complementarity between AAT and TMPRSS2 is poor to moderate at both the buried interface and the solvent-exposed interface rim (Figs. 6D and S3A,B). Notably, positively-charged regions of AAT and TMPRSS2 at the interface rim are predicted to be unfavorably close (Figs. 6E and S3A,B dotted ellipse). The charge complementarity between the sequence of the AAT RCL and the negatively-charged protease active site is also low, especially at the critical P1 position of AAT (Fig. 6F). However, hydrogen-bonding interactions between the RCL and the protease active site in TMPRSS2–AAT are identical to those of trypsin–AAT (Figs. 6C and S3C,D). We also modeled the complex between TMPRSS2 and a recently identified endogenous TMPRSS2 inhibitor, HAI-2^21^, using the structure of a mesotrypsin–HAI-2 complex. Structural comparison revealed a remarkable agreement between the RCL of AAT and the inhibitory loop of HAI-2 (Fig. S4), further validating the reliability of our analyses.

To better understand the structural modelling results, we calculated binding free energies for all protease-inhibitor complexes used in this study (Table 1). The modeled TMPRSS2–AAT complex has the poorest predicted binding affinity, consistent with the unfavorable electrostatic interactions at the interface (Figs. 6D–F and S3A,B).

**Table 1:** Predicted binding free energies for representative protease-inhibitor complexes.

Complex	PDB ID	Binding free energy (Δ*G*)/ kcal/mol^a^
Bovine cationic S195A trypsin—human AAT Pittsburgh complex	1OPH	− 131.0
Human mesotrypsin—HAI-2 kunitz domain 1 inhibitor complex	4U32	− 125.7
TMPRSS2—HAI-2 complex	–	− 114.0
TMPRSS2—AAT complex	–	− 110.8

^a^Calculations were performed using the Molecular Mechanics-Generalized Born and Surface Area (MM-GBSA) approach in BioLuminate (version 1.0, Schrödinger, LLC, New York, NY, 2012). Ranked according to Δ*G:* a more negative value indicates stronger binding.

Given the ability of heparin to augment AAT inhibition of TMPRSS2, we performed heparin docking calculations using the TMPRSS2–AAT model. Docking heparin tetrasaccharides revealed binding exclusively at a major binding hotspot on the solvent-exposed surface at the rim of the TMPRSS2–AAT interface, extending approximately three quarters of its circumference (Figs. 6D,E and S5A). Docking analysis suggests that heparin molecules may act as electrostatic bridges between AAT and TMPRSS2, likely stabilizing an otherwise suboptimal complex and thus enhancing AAT inhibition of TMPRSS2. More specifically, heparin appears to bind at several clusters of positively charged amino acid residues at the interface edge, neutralizing otherwise repulsive forces (Fig. 6D,E).

The calculated molecular weight (MW) of the TMPRSS2 protein is 53.9 kDa, less than that indicated in Figs. 1B,C. This discrepancy is likely due to several factiors including that MW markers on SDS-PAGE gels provide only an estimate of the MW and the need to add an additional ~ 5 kDa to TMPRSS2 for the ~ 6 histidine-tagged residues plus potential glycosylation at N213 and N249 as calculated by GlyConnect (https://glyconnect.expasy.org/browser/proteins/1848^22^). Inspection of glycosylation sites in TMPRSS2 (N213 and N249) and AAT (N46, N83, and N247) reveals that all are distant from heparin-bridging regions, indicating that glycosylation is unlikely to impact heparin binding (Fig. S5B).

Calculation of electrostatic surfaces of an elastase–AAT complex shows high charge complementarity between the two proteins (Fig. S6A,B), consistent with the observed lack of additional effect of any of the heparins on AAT inhibition of elastase activity (Figs. S1 and S2). Taken together, modeling reveals how the RCL of AAT adopts an inhibitory-competent conformation, and how low to moderate charge complementarity between TMPRSS2 and AAT is partially rescued by the binding of heparin molecules, which act as bridges between positively charged residues at the rim of the complex interface. Thus, these results provide a structural rationalization for the ability of enoxaparin to enhance AAT inhibition of TMPRSS2.

## Discussion

Effective vaccines are available and are essential to quell the COVID-19 pandemic. However, vaccine-refusal in 25–30% of the U.S. population^23^, fatigue in public health measures such as masking, and evolution of highly infectious strains of SARS-CoV-2 (*e.g.*, the B.1.617.2 delta strain and the more recent Omicron and its subvariant strains) likely account for the resurgence of cases. Breakthrough cases in vaccinated individuals have also occurred^24^. Furthermore, some vaccines are less protective against the delta strain and the increasingly dominant Omicron subvariant strain, which is also more resistant to neutralizing antibodies^25,26^.

In the context of these sobering issues, optimizing treatment for severe COVID-19 is a priority but remains suboptimal. While an expansive array of agents have been studied against severe COVID-19, only a few have shown efficacy and modest at best. Dexamethasone remains the most widely used agent for those with severe COVID-19 and has been shown in the RECOVERY study to have an absolute reduction in mortality of 3.5% to 11.7% in those with severe COVID-19^27^. Anti-IL6 receptor antibodies (*e.g.*, tocilizumab) or an anti-Janus kinase (JAK) inhibitor (baricitinib) is recommended as adjunct to dexamethasone in critically-ill patients, especially if they remain recalcitrant to dexamethasone^28^. Recently, however, the FDA has issued a Black Box warning for anti-JAK agents because of risks of serious cardiac events and cancer. Although a double-blind, placebo-controlled study showed that remdesivir therapy reduced the recovery time from COVID-19 by ~ 4 days and showed a trend toward improved mortality^29^, an extensive analysis showed that remdesivir did not affect on the use of mechanical ventilation, length of hospital stay, or mortality^30^. Host-directed therapy, as proposed herein, is less likely to be affected by SARS-CoV-2 mutations and has the potential to target both the viral infection and the multiple pathogenic mechanisms responsible for severe COVID-19^3^.

While we and others found that AAT inhibits TMPRSS2 activity in a physiologic, dose-dependent fashion in the context of SARS-CoV-2 infection^2,7^, it is important to note that over 5 years ago, AAT was reported to inhibit TMPRSS2, which is also required to activate hepatitis C virus infection^31^. However, to the best of our knowledge, we are the first to report that enoxaparin significantly augmented AAT inhibition of TMPRSS2 activity and an in vitro infection with a human coronavirus. Highly rigorous model building provided a structural rationalization for the ability of enoxaparin to augment AAT inhibition of TMPRSS2. Although AAT can form very similar interactions with the active site of TMPRSS2 compared to that with trypsin, charge complementarity at the interface, especially the S1 pocket, is much lower. Therefore, it is likely that their association is weakened, lowering the inhibitory potency of AAT. We hypothesize that heparins, especially enoxaparin, augment AAT inhibition of TMPRSS2 activity by acting as a bridge, stabilizing unfavorable charge-charge interactions and thus improving the molecular compatibility at the complex interface. This functional effect is consistent with the known importance of electrostatics in forming AAT–protease interactions^32^ and the role of glycosaminoglycans in facilitating protease–serpin complex formation; *i.e.*, heparin acts as a template for binding protease and serpin, creating a more stabilized ternary complex^33,34^.

There are several well-documented examples of serpins inhibiting membrane-bound proteases^35^. In such cases, including TMPRSS2, the juxtaposition of the protease and the membrane is predicted not to interfere with the inhibitory mechanism of the serpin. On the contrary, the serpin mechanism of forming an irreversible linkage with the protease may in fact facilitate protease removal and thus regulation of proteolytic activity by direct binding between the serpin and the membrane-bound endocytic receptor LDL receptor–related protein (LRP)^36^.

**Figure 7:**
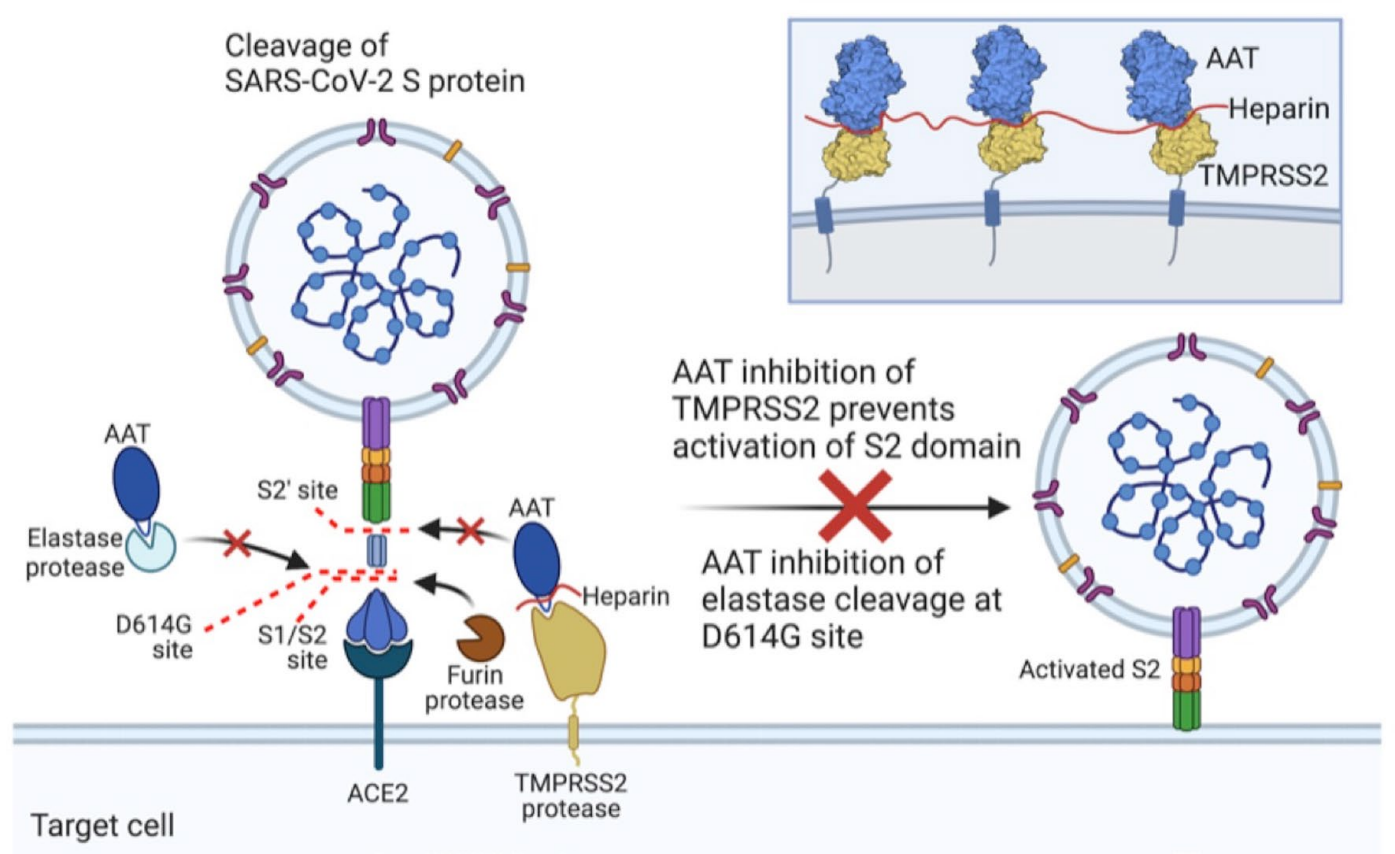
Proposed model for TMPRSS2–AAT–heparin ternary complexation at the cell surface, preventing activation of the S2 domain. Heparin (red) neutralizes the repulsive electrostatic forces at the TMPRSS2 (wheat)–AAT (blue) interaction rim, in accordance with the predicted binding mode shown in Figs. 6D,E and S5. AAT inhibition of elastase cleavage at the site created by the D614G mutation of the spike protein (B.1.1.7 variant of SARS-CoV-2) is also shown. The inset shows the possibility that one heparin molecule could bridge multiple TMPRSS2–AAT complexes.

Although all heparins are anionic polysaccharides, the products are comprised of a mixture of different chain lengths of sulfated glycosaminoglycans. Whereas the average MW of UFH is 15,000 Da, the range is estimated to be 3 to ≥ 30 kDa^37^. In contrast, the average MW of enoxaparin and nadroparin is much lower—estimated to be 4300 and 4500 Da, respectively. Yet, even low-molecular weight heparin—isolated from UFH by various methods of fractionation or depolymerization—is comprised of glycosaminoglycan chains of varying lengths. While the affinity of AAT binding to TMPRSS2 is modest based on molecular modeling, this interaction may be enhanced in vivo because the plasma level of endogenous UFH is 1 to 2.4 mg/L, equivalent to 0.5 to 1.2 units/mL^38^. While our model showed that a 4mer of negatively-charged heparin acts as a bridge for the electropositive patches at the TMPRSS2–AAT interface, several such sites may be occupied by the binding of a heparin molecule much longer than a tetrasaccharide, extending to other positively-charged sites on the TMPRSS2–AAT complex (Fig. 7, inset). The mode of heparin bridging in the TMPRSS2–AAT complex resembles that proposed for the dextran–C1s–C1INH ternary complex, whereby one dextran sulfate molecule can bridge multiple C1s–C1INH complexes^9^. Accordingly, a proposed mechanism for heparin–TMPRSS2–AAT engagement is shown in Fig. 7.

AAT is normally glycosylated with negatively charged sialic acid residues^39^, which may interfere with heparin binding. However, our modeling showed that the sites where the heparin appears to bridge the TMPRSS2–AAT complex are distant from these glycosylated sites. Indeed, glycosylation may provide one plausible explanation for the greater enhancement by the smaller enoxaparin compared with UFH; *i.e.*, the presence of bulky sugars on AAT is likely to obstruct the binding of longer polysaccharides than of shorter molecules. The fact that the other low-molecular weight heparin (nadroparin) did not augment AAT inhibition of TMPRSS2 suggests the possibility that specific lengths of heparin are optimal in this bridging. In addition, the spatial orientation and density of TMPRSS2 at the cell surface may have implications for optimal heparin lengths (Fig. 7, inset).

In the U.S., it is estimated that there are ~ 300,000 individuals with significant AAT deficiency, most often caused by the protease inhibitor (Pi) ZZ genotype. Given our findings and the previously mentioned studies showing that those with AAT deficiency are more predisposed to severe disease and death from COVID-19, it is reasonable to surmise that AAT augmentation in those with severe AAT deficiency would be beneficial against COVID-19. Nevertheless, even in the absence of frank AAT deficiency, the AAT response to a systemic infection may be inadequate as has been shown for hospitalized COVID-19 patients^40^ and among millions with certain heterozygous AAT mutations^41^. This possibility leads to the question of whether AAT supplementation would be beneficial even in those without frank AAT deficiency? Indicative of its homeostatic function, AAT levels often increase several fold with systemic inflammation and/or infection^42^. Thus, in individuals who are unable to mount an adequate AAT response to a severe infection, whether due to genetic or epigenetic causes, AAT supplementation may potentially be helpful. While elevated levels of AAT found in plasma would be expected to counter SARS-CoV-2 viremia, the lower levels normally found in the airway lining fluid leads us to speculate that exogenous AAT may have additional benefit in certain individuals without frank AAT deficiency. This notion is supported by murine studies wherein exogenous AAT given to mice not deficient in endogenous AAT (or a transgenic mouse the overexpresses AAT) provided further protection against various infectious agents and inflammatory disorders^43–47^. Furthermore, oxidation of methionine 351 and/or 358 of even normal AAT may cause loss of its serpin activity^48^. Since COVID-19 is associated with increased oxidative stress^49^, the anti-protease activity of AAT may be rendered ineffective even with an adequate response.

AAT has a panoply of biological and potentially therapeutic functions against COVID-19 that are only in part dependent on its serpin activity and for which the dose-responses are unknown. In addition to inhibiting TMPRSS2, we had posited six other mechanisms by which AAT may ameliorate COVID-19^3^: *(i)* induction of autophagy, known to protect against the coronavirus that caused the Middle East Respiratory Syndrome; *(ii)* antagonism of inflammation; *(iii)* inhibition of neutrophil elastase, an important mediator of acute lung injury; *(iv)* inhibition of thrombin; *(v)* protection against COVID-19-associated endothelial injury*;* and *(vi)* alteration in the formation of neutrophil extracellular traps (NETs)—comprised of extracellular DNA decorated with elastase, cathepsin G, and histones, and are implicated in the immunothrombosis of COVID-19^50^. Two mechanisms by which AAT could enhance autophagy—a plausible mechanism by which intracellular SARS-CoV-2 could be destroyed—is direct inhibition of NFκB activation^51–53^ and the ability of AAT to decrease translocation of the components of NADPH oxidase from the cytoplasm to the plasma membrane resulting in decreased production of reactive oxygen species (ROS)^54^. Since ROS can activate NFκB and we have previously shown that inhibition of NFκB can induce autophagic flux^55^, the denouement of this function of AAT is to enhance autophagy by inhibiting both formation of ROS and activation of NFκB. One caveat to this hypothesized paradigm is that while AAT can inhibit ROS formation, ROS can, in turn, inactivate AAT. Perhaps the increased AAT levels seen with systemic inflammation and infection (and maybe the host-directed antimicrobial effects of pharmacologic doses of AAT in animal models) serve to favor AAT inhibition of ROS formation over the reverse reaction^43^. In addition, the excessive inflammation reported with severe COVID-19 may augment sialylation of AAT, resulting in increased binding to IL-8, depriving a key chemokine for tissue-damaging neutrophils^39,56^. de Loyola and co-workers^57^ also showed that AAT inhibits disintegrin/metalloproteinase 17 (ADAM17, a sheddase of some cytokines), which would also have an anti-inflammatory effect. AAT also impedes the cellular entry of the D614G variant of SARS-CoV-2 by inhibiting elastase cleavge of a mutated site on the spike protein, also known to activate this B.1.1.7 lineage^58^ (Fig. 7).

There are other potential mechanisms by which the heparins themselves may mitigate against COVID-19 other than its anti-TMPRSS2 or anticoagulant effect, including: *(i)* competively inhibiting cell surface heparan sulfate, a co-receptor SARS-CoV-2^59^, *(ii)* anti-inflammatory effect by binding to pro-inflammatory molecules (IL-8, major basic protein, and complement components), inhibiting nuclear factor-kappa B activation, and reducing the release and activity of IL-6; *(iii)* binding to extracellular histones released from dead cells, mitigating histone-mediated endothelial and organ dysfunction; and *(iv)* interacting with endothelial cells to maintain vascular integrity^60,61^.

In summary, we discovered biochemical, biological, and structural modeling evidence that enoxaparin enhances AAT inhibition of TMPRSS2 and a human coronavirus infection of hAEc. Furthermore, because of the panoply of other activities that both AAT and heparin possess against the multitude of pathophysiologic processes associated with severe COVID-19, we believe these findings provide a rational basis for studying the combination of AAT and enoxaparin—both of which have a strong safety record and are currently in use for other indications—for critically ill patients with COVID-19 to limit progressive disease and death.

## Methods

### Materials

The human cell line HEK293T (CRL-3216™), human coronavirus 229E (HCoV-229E), and VeroE6 cells (CRL-1586™) were obtained from the American Type Culture Collection (Manassas, VA). Alpha-1-antitrypsin (AAT, Glassia^®^) was acquired from Takeda Pharmaceuticals, Lexington, MA. The remaining key reagents and materials as well as overexpression of TMPRSS2 in HEK293T cells, TMPRSS2 activity assay, elastase assay, infection with HCoV-229E, plaque assay, and more standardized assays are in the Supplemental Information.

### Overexpression of TMPRSS2 in HEK293T cells

A pcDNA3.1 plasmid containing both the human TMPRSS2 open reading frame and the neomycin resistance gene was obtained from Addgene (Watertown, MA). This plasmid was sub-cloned with a C-terminal His Tag, designated as pcDNA3.1^TMPRSS2+His^ (Fig. 1A, top panel). The empty vector (pcDNA3.1) and pcDNA3.1^eGFP^ plasmid were used as controls.

HEK293T cells were grown in DMEM supplemented with 10% FBS, 2 mM l-glutamine and 100 units/mL penicillin–streptomycin and seeded in a 6-well plate (1–2 × 10^5^ cells/well) or a black-bottom 96-well plate (8.5 × 10^4^ cells/well). Following overnight incubation at 37 °C and 5% ambient CO_2_, the cells were transfected for 2 days with either the empty vector pcDNA3.1 or pcDNA3.1^TMPRSS2+His^ plasmid via Lipofectamine^®^ according to the manufacturer’s protocol. The pcDNA3.1^eGFP^ plasmid was used to monitor transfection efficiency. The cells that were transfected with the pcDNA3.1 or pcDNA3.1^TMPRSS2+His^ plasmid were selected with neomycin.

### Measurement of TMPRSS2 activity

HEK293T cells that overexpress TMPRSS2 (HEK293T^TMPRSS2^) were left untreated or pre-treated with AAT (1, 3, and 5 mg/mL), UFH (1.5 and 8 U/mL), enoxaparin (35 and 70 μg/mL), nadroparin (2.2 and 8.8 U/mL), and/or AEBSF (50 μM) and incubated at 25 °C for 30 min, followed by the addition of 100 µM of the fluorogenic substrate Boc-QAR-AMC. Fluorescence was immediately measured using a UV filter (excitation 365 nm and emission 410 nm) and then every 15 min for a total of 90 min at 37 °C in a SpectraMax M2e Microplate Reader (Molecular Devices LLC) and reported as arbitrary fluorescent units (AFU).

### Culture of primary human airway epithelium in air–liquid interface

Primary human airway tissue was isolated from human lungs procured with consent from with consent from legally authorized representatives by the Human Lung Tissue Consortium at National Jewish Health under National Jewish Health IRB protocol HS-3209. Briefly, lungs are accepted from deceased donors free of lung disease who died of non-pulmonary causes. Organs are transported to the research site in either Histidine-tryptophan-ketoglutarate (HTK) or University of Wisconsin (UW) transplant solutions while kept on ice. Organs are processed for end users immediately upon arrival and always within 24 h of harvest.

Air–liquid interface (ALI) culture of primary human airway epithelial cells (hAEc) from a healthy, cadaveric lung donor were prepared as previously reported^62^. In brief, basal stem cells isolated from the cadaveric lungs were expanded on Collagen I-coated plastic dishes in Pneumacult Ex + medium and cryopreserved. From these existing repository cells, freshly thawed cells were seeded on Collagen I-coated Transwell inserts at a concentration of 1 × 10^5^ cells/cm^2^. Cells initially proliferate to confluency under submerged conditions in Pneumacult Ex + medium, then are transitioned to an ALI by supplying Pneumacult ALI medium only from below the insert. Cells differentiate into a mature mucociliary epithelium over 21 days of ALI culture, which faithfully models the in vivo mucosal cellular characteristics. Primary hAEc grown in ALI express both TMPRSS2 and to a lesser extent ACE2^63^.

### Infection of hAEc with human coronavirus 229E

After culture and differentiation of hAEc grown on ALI for 3–4 weeks, the medium was replaced with fresh medium alone or pre-treated for 1 h with medium containing AAT (3 mg/mL), enoxaparin (70 μg/mL), or both in both the apical and bottom chambers of the 24-well Transwell plate, followed by infection with human coronavirus 229E (HCoV-229E) at an multiplicity-of-infection of 1 hAEc:0.01 HCoV-229E in the apical chamber. HCoV-229E is an instructive viral challenge because the virus also uses TMPRSS2 to process its spike protein. Unlike other less virulent human coronaviruses, HCoV-229E has been associated with respiratory failure manifested as the acute respiratory distress syndrome^64^. In addition, since our focus was to study the effects of AAT on viral infection in the context of AAT inhibition of TMPRSS2, we utilized a coronavirus which uses a different receptor (aminopeptidase N rather than ACE2) since AAT has been shown to inhibit ADAM17, which induces shedding of ACE2 from the cell surface. After two hours of incubation for viral adsorption, the cells in the apical chamber were washed with wash buffer (PBS:medium 1:1). After incubation at 37 °C (5% CO_2_) for 3 days, 150 μL of the medium was added to each apical chamber, incubated for 30 min, and the supernatant in the apical chamber was pipetted gently to recover the viral particles. The hAEc seeded on the transwells were also saved for viral protein immunostaining. All samples were stored at − 80 °C until assayed.

### Immunocytofluorescent analysis of HCoV-229E-infected primary hAEc

To quantify the viral load of the cells using an alternative method, we performed immunofluorescent staining of the infected hAEc for the nucleoprotein of HCoV-229E. The membranes of the Transwell inserts were cut, placed upside down on a glass slide, and cytocentrifuged at 1850 rpm for 5 min to dissociate the cells from the membrane onto the glass slide. The cells on the glass slide were then fixed with 4% paraformaldehyde in PBS at 4 °C for overnight. The fixed hAEc were treated with permeabilized buffer (0.5% Triton-X diluted in 1× PBS) for 10 min, rinsed thrice with PBS, and blocking buffer (5% BSA and 0.5% Tween20 in 1× PBS) added for one hour. Then the fixed cells were incubated with an anti-nucleocapsid polyclonal antibody (1:100) directed against the nucleoprotein/NP of HCoV-229E in blocking buffer at 4 °C overnight with gentle shaking. After washing three times, Cy3-tagged anti-rabbit antibody (1:1000) was added for two hours, following by mounting with ProBong Gold Antifade reagent with DAPI. All cellular images were viewed as a single plane using a fluorescent microscope (Carl Zeiss Anxiovert 200M).

### Plaque assay

A total of 2 × 10^6^ VeroE6 cells were seeded in 6-well plates for 2 days at 37 °C and 5% CO_2_ until 100% confluent growth. Before inoculation, the medium was removed and washed with PBS. The supernatant containing HCoV-229E from infected hAEc was diluted and inoculated onto the VeroE6 cells and incubated at 35 °C for 1–1.5 h with brief rocking every 15 min. The cell monolayers were then overlayed with two mL of 0.8% methylcellulose (Sigma M0512) in DMEM + 10% FBS, 100 U/mL streptomycin/penicillin, 2 mM l-glutamine, and incubated at 35 °C for 4 days. The methylcellulose was then removed and the cells were fixed with 4% paraformaldehyde overnight at room temperature and stained with 0.2% Crystal Violet dissolved in 10% of ethanol and 90% of PBS. After washing, the plate was air dried. Plaque forming units (PFU)/mL were determined based on the formula PFU/mL = number of plaques/(D × V), where D = dilution factor, V = volume of diluted virus/well.

### Molecular modeling and computational methods

#### Construction of full-length TMPRSS2 model

Sequence analysis of TMPRSS2 (UniProtKB ID O15393 (TMPS2_HUMAN)) revealed the presence of three extracellular domains: an N-terminal low-density lipoprotein receptor A (LDLR-A) domain, followed by a scavenger receptor cysteine-rich repeat (SRCR) domain, and finally a catalytic peptidase S1 domain. The crystal structure of human TMPRSS2 in complex with the inhibitor Nafamostat (PDB ID 7MEQ) consists of SRCR and S1 domains only—the LDLR-A domain is too flexible to be modeled into electron density^20^. However, a high-quality model of full-length TMPRSS2 was made available by prediction from AlphaFold, a powerful artificial intelligence program by DeepMind (https://alphafold.ebi.ac.uk/entry/O15393)^65^. A composite model consisting of SRCR and S1 domains from 7MEQ and the LDLR-A domain from the AlphaFold model was constructed. Loop regions that are missing in the TMPRSS2 crystal structure (164–166; 203–207; 217–220) were modelled using the *Rosetta Remodel* protocol^66^. All other regions of the SRCR and S1 domains are identical to the structure of 7MEQ.

Verification of side-chain rotamers and intramolecular contacts was performed using *Coot*^67^, followed by structure refinement using the *Rosetta Relax* protocol^68^. The final model consists of residues 109–491, corresponding to a near 100% complete extracellular sequence, except four N-terminal residues 106–108 which were not modeled.

#### Modeling of TMPRSS2–AAT Michaelis complex

A TMPRSS2–AAT Michaelis complex model was constructed using bovine cationic S195A trypsin—human AAT Pittsburgh Michaelis complex (M358R; PDB ID 1OPH)^69^. TMPRSS2 was structurally aligned to trypsin, and P1 methionine (residue 358) of AAT was mutated back to arginine in *Coot* to generate the wildtype sequence*.* After manual inspection, the model was subjected to refinement using the *Rosetta Relax* protocol^68^.

#### Modeling of TMPRSS2–HAI-2 complex

The structure of a complex between human mesotrypsin and HAI-2 kunitz domain 1 inhibitor (PDB ID 4U32) was used to model a TMPRSS2–HAI-2 complex. TMPRSS2 was first structurally aligned with the protease chain in the mesotrypsin-HAI-2 complex and then combined with HAI-2 coordinates. The complex was then manually inspected in *Coot* and subjected to refinement using *Rosetta Relax*.

#### Heparin docking

Heparin docking was performed using *ClusPro*^70^ and a 4mer heparin molecule. Docked solutions were ranked according to cluster size. Structural representations were produced using Schrodinger *PyMOL version 2.5.1*. Electrostatic calculations were performed with the *APBS* plugin^71^ in PyMOL. Structural alignments were performed using *MUSTANG-MR*^72^*.*

#### Protein–protein binding energy calculations

The binding free energies (Δ*G*) for protease-inhibitor complexes was performed using the Molecular Mechanics-Generalized Born and Surface Area (MM-GBSA) approach^73^ in BioLuminate (version 1.0, Schrödinger, LLC, New York, NY, 2012), the OPLS2005 force field^74,75^ and VSGB solvent model^76^. Δ*G* is calculated according to the equation:

$$\Delta G = {\text{ E}}\_{\text{complex}}\left( {{\text{minimized}}} \right) - {\text{ E}}\_{\text{ligand}}\left( {{\text{minimized}}} \right) - {\text{E}}\_{\text{receptor}}\left( {{\text{minimized}}} \right)$$

The absolute values calculated are not necessarily in agreement with experimental binding affinities. However, the ranking based on the calculated binding energies can be expected to agree reasonably well with ranking based on experimental binding affinity.

## Supplementary Information

Supplementary Information.

## Data Availability

Model coordinates have been deposited in the ModelArchive database: TMPRSS2–AAT Michaelis complex (https://modelarchive.org/doi/10.5452/ma-iwo48); TMPRSS2–HAI-2 complex (https://modelarchive.org/doi/10.5452/ma-xw38b).
