# Author Correction: Enoxaparin augments alpha-1-antitrypsin inhibition of TMPRSS2, a promising drug combination against COVID-19

**DOI:** 10.1038/s41598-022-10890-w

**Published:** 2022-04-19

**Authors:** Xiyuan Bai, Ashley M. Buckle, Eszter K. Vladar, Edward N. Janoff, Reeti Khare, Diane Ordway, David Beckham, Lorelenn B. Fornis, Abraham Majluf-Cruz, Randolph V. Fugit, Brian M. Freed, Soohyun Kim, Robert A. Sandhaus, Edward D. Chan

**Affiliations:** 1 Department of Medicine, Rocky Mountain Regional Veterans Affairs Medical Center, Aurora, CO USA; 2 Department of Pharmacy, Rocky Mountain Regional Veterans Affairs Medical Center, Aurora, CO USA; 3 Department of Academic Affairs and Medicine, National Jewish Health, Denver, CO USA; 4 Mycobacteriology Laboratory, Advance Diagnostics, National Jewish Health, Denver, CO USA; 5 Division of Pulmonary Sciences and Critical Care Medicine, University of Colorado Anschutz Medical Campus, Aurora, CO USA; 6 Division of Infectious Diseases, University of Colorado Anschutz Medical Campus, Aurora, CO USA; 7 Department of Immunology, University of Colorado Anschutz Medical Campus, Aurora, CO USA; 8 Department of Microbiology, Immunlogy, and Pathology, Colorado State University, Fort Collins, CO USA; 9 Department of Biochemistry and Molecular Biology, Biomedicine Discovery Institute, Monash University, Clayton, VIC Australia; 10 Laboratory of Cytokine Immunology, Department of Biomedical Science and Technology, Konkuk University, Seoul, South Korea; 11 College of Veterinary Medicine, Konkuk University, Seoul, South Korea; 12 Unidad de Investigacion Medica en Trombosis, Hemostasia y Aterogenesis, Instituto Mexicano del Seguro Social, Mexico City, Mexico; 13 National Jewish Health, D509, Neustadt Building, 1400 Jackson Street, Denver, CO 80206 USA

Correction to: *Scientific Reports* 10.1038/s41598-022-09133-9, published online 25 March 2022

The original version of this Article contained an error in the x-axis labels of Figure 5B, C, where in Figure 5B, the spacing of "−" and "+" indicators was incorrect and in Figure 5C, the "−" and "+" indicators were missing.

The original Figure 5 and accompanying legend appear below.

**Figure 5:**
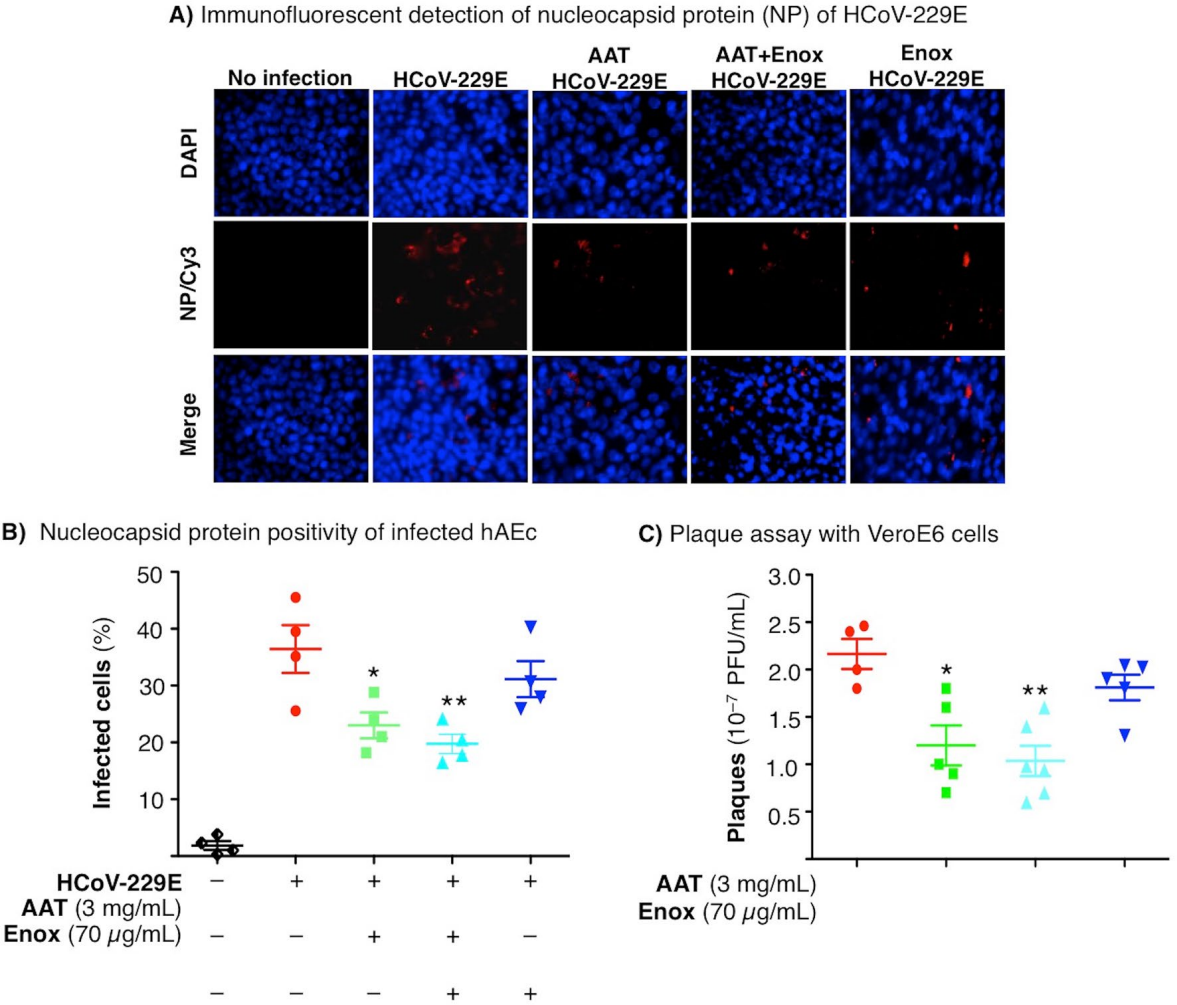
Effects of AAT, enoxaparin, or both on infection of hAEc with HCoV-229E. (**A**) Immunofluorescence analysis of HCoV-229E-infected hAEc grown in air–liquid interface. The hAEc were pre-treated with AAT (3 mg/mL), enoxaparin (70 µg/mL), or both for 1 h, and then infected with HCoV-229E at a multiplicity-of-infection of 1 hAEc:0.01 HCoV-229E. Three days after infection, the cells were fluorescently immunostained for the nucleocapsid protein of HCoV-229E. The nuclei were stained with DAPI. Fluorescent images were taken at a magnification of 400X by confocal microscopy (Carl Zeiss Anxiovert 200 M). (**B**) Percentage of HCoV-229E-infected hAEc with > 700 total cells counted for each condition. Data shown are the mean ± SEM of three independent experiments. *p < 0.05, **p < 0.01 compared to HCoV-229E infection alone. (**C**) Plaque assay. The apical chamber medium of HCoV-229E-infected hAEc were used to infect VeroE6 cells and incubated for 4–5 days. Infection of the hAEc were done in triplicates and subsequent infection of VeroE6 cells with the supernatant of HCoV-229E-infected hAEc were each done in triplicates. Thus, the data shown are the triplicate means ± SEM of the original triplicate experiments. *p < 0.05 and **p < 0.01 compared to cells infected with HCoV-229E only without treatment. hAEc = primary human airway epithelial cells, HCoV-229E = human coronavirus 229E.

The original Article has been corrected.

