# Supplementary material for: Enoxaparin augments alpha-1-antitrypsin inhibition of TMPRSS2, a promising drug combination against COVID-19
Source: Sci Rep. 2022 Mar 25;12:5207. doi: 10.1038/s41598-022-09133-9 (PMC8953970; doi:10.1038/s41598-022-09133-9)
Supplement: Supplementary file 1 — Supplementary Information. [file 41598_2022_9133_MOESM1_ESM.pdf]

Enoxaparin augments alpha-1-antitrypsin inhibition of TMPRSS2, a promising drug combination against COVID-19

SUPPLEMENTARY INFORMATION: Contains Methods, full length immunoblots for Figure 1B and 1C, and Six Supplementary Figures

Xiyuan Bai, Ph.D.[1,3,5]
Ashley M. Buckle, Ph.D.[9]
Eszter K. Vladar, Ph.D.[5]
Edward N. Janoff, M.D.[1,6]
Reeti Khare, Ph.D.[4]
Diane Ordway, Ph.D.[8]
David Beckham, M.D.[6]
Lorelenn B. Fornis[3]
Abraham Majluf-Cruz, M.D.[12]
Randolph V. Fugit, Pharm.D.[2]
Brian M. Freed, Ph.D.[7]
Soohyun Kim, Ph.D.[10,11]
Robert A. Sandhaus, M.D., Ph.D.[3]
Edward D. Chan, M.D.[1,3,5,*]

[1]Department of Medicine, [2]Department of Pharmacy, Rocky Mountain Regional Veterans Affairs Medical Center, Aurora, Colorado, [3]Departments of Academic Affairs and Medicine, [4]Mycobacteriology Laboratory, Advance Diagnostics, National Jewish Health, Denver, Colorado, [5]Division of Pulmonary Sciences and Critical Care Medicine, [6]Division of Infectious Diseases, [7]Department of Immunology, University of Colorado Anschutz Medical Campus, Aurora, Colorado, [8]Department of Microbiology, Immunlogy, and Pathology, Colorado State University, Fort Collins, Colorado, [9]Department of Biochemistry and Molecular Biology, Biomedicine Discovery Institute, Monash University, Clayton, Victoria, Australia, [10]Laboratory of Cytokine Immunology, Department of Biomedical Science and Technology; [11]College of Veterinary Medicine, Konkuk University, Seoul, Korea, [12]Unidad de Investigacion Medica en Trombosis, Hemostasia y Aterogenesis, Instituto Mexicano del Seguro Social, Mexico City, Mexico.

Xiyuan Bai and Ashley M. Buckle contributed equally to the project

Running head: Enoxaparin augments AAT inhibition of TMPRSS2 activity and human coronavirus 229E infection

*__Addresses correspondence to__:
Edward D. Chan, M.D.
D509, Neustadt Building
National Jewish Health
1400 Jackson Street
Denver, CO 80206
Phone 303-398-1491, Fax: 303-270-2185
E-mail: ChanE@NJHealth.org

Methods

Materials

The human cell line HEK293T (CRL-3216^{TM}), human coronavirus 229E (HCoV-229E), and VeroE6 cells (CRL-1586^{TM}) were obtained from the American Type Culture Collection (Manassas, VA). Alpha-1-antitrypsin (AAT, Glassia®) was acquired from Kamada Ltd., Israel. AEBSF and Boc-Gln-Ala-Arg-7-amino-4-methylcoumarin (Boc-QAR-AMC fluorogenic peptide substrate) were purchased from R&D Systems (Minneapolis, MN). Unfractionated heparin sodium (1,000 units/mL) was obtained from Fresenius Kabi, Lake Zurich, IL. Enoxaparin sodium (100 mg/mL) was obtained from Winthrop, Bridgewater, NJ. Nadroparin (Fraxiparine, 950 IU of anti-Xa per 0.1 mL) was obtained from Aspen Holdings Pharmaceutical, Durban, South Africa. Fetal bovine serum (FBS) was purchased from Atlanta Biologicals (Norceross, GA) and inactivated at 56°C for one hour. DMEM medium, L-glutamine, penicillin-streptomycin, polyclonal anti-TMPRSS2 antibody, 293 SFM II (serum-free medium), and the Lipofectamine® Reagent were purchased from ThermoFisher Scientific/Life Technologies (Carlsbad, CA). The Transwell tissue culture plate (with 0.4 µm Pore Polyester Membrane Insert, #3470) to culture human airway epithelial cells in air-liquid interface was purchased from Corning Corporation, Glendale, AZ. Cy3-goat anti-rabbit IgG (H +L), Lab-Tek II Chamber Slide system, polyclonal rabbit-human antibody β-actin, His-Tag, and Phototope-HRP Western Blot Detection System were purchased from Cell Signaling Technology (Danvers, MA). The EnzChek® Elastase Assay Kit was purchased from Molecular Probes, Eugene, OR. The anti-nucleocapsid polyclonal antibody directed against the nucleoprotein of HCoV-229E was purchased from Sino Biological Inc (Cat No. 40640-T62).

Concentrations of UFH, enoxaparin, and nadroparin tested

A standard initial dose of UFH given to patients is 5,000 to 10,000 units subcutaneously or intravenously to prevent or treat venous blood clots. Maintenance doses are then given.

Assuming UFH (5,000 or 10,000 units) is given intravenously and the average plasma volume is 3 liters, the plasma concentration would approximate 1.7 to 3.4 units/mL. Thus, the UFH concentrations used were 0, 1.5, and 8 units/mL. For the standard enoxaparin dose of 1.5 mg/kg, calculations based on a 70 kg person and 3 liters of plasma gives an enoxaparin concentration of 35 μg/mL. Thus, the final enoxaparin concentrations used were 0, 35, and 70 μg/mL. Similarly for nadroparin, a 0.1 mL/kg dose of a 5,700 units per 0.6 mL solution gives an estimated plasma concentration of 2.2 units/mL. Thus, the final nadroparin concentrations used were 0, 2.2, and 8.8 units/mL.

Western blot

HEK293T cells transfected with pcDNA3.1 or pcDNA3.1$^{TMPRSS2+His}$ were lysed with cell lysis buffer (Cell Signaling Technology). Loading buffer (Life Technologies) was added, the mixture heated to 98°C for 5 min, and 30 μg of protein lysates for each sample were separated by SDS-PAGE using 12% NuPAGE Bis-Tris Gels (Life Technologies). The proteins were transferred to nitrocellulose membranes by iBot2 instrument, blocked with 5% milk, and probed with either monoclonal anti-His antibody (1:1000) (Bio-Rad), polyclonal anti-TMPRSS2 antibody (1:1000), or anti-human β-actin (1:3000), and subsequently with HRP-conjugated anti-mouse IgG or anti-rabbit IgG secondary antibody in a 1:2000 dilution. The immunoblots were visualized by chemiluminescence using HPR western blot system (Cell Signaling Technology).

Immunocytochemistry

HEK293T cells transfected with pcDNA3.1 or pcDNA3.1$^{TMPRSS2+His}$ were seeded in Nunc Lab-Tek II chamber slides and cultured for two days. The cells were then washed with a 1:1 solution of DMEM medium:1X phosphate buffer saline (PBS), fixed with 4% paraformaldehyde for 30 minutes at room temperature, rinsed with 1X PBS, and then permeabilized with 0.5% Triton X-100 in PBS for 10-20 minutes. The cells were then sequentially incubated with

blocking buffer for 1-2 hours, rabbit anti-TMPRSS2 antibody (1:100) overnight at 4°C, and Cy3-labeled goat anti-rabbit IgG (H+L) secondary antibody (1:1000) at room temperature for 1-2 hours. The slides were sealed with ProLong Gold Antifade Mountant with DAPI (ThermoFisher Scientific) and analyzed at 400X magnification using a fluorescence microscope (Carl Zeiss Axiovert 200M) equipped with DAPI and Cy3 filters.

Following infection of human airway epithelial cells with human airway epithelial cells (hAEc) with human coronavirus 229E, the cells were fluorescently immunostained with anti-nucleocapsid protein polyclonal antibody (1:100) and goat Cy3-tagged anti-rabbit IgG (H+L) (1:1000).

Elastase Assay

Elastase activity was measured according to the manufacturer's instructions. In brief, solutions of porcine pancreatic elastase, DQ^{TM} elastin substrate labeled with a quenched fluorescent dye, AAT, three formulations of heparin, and AAT plus each of the heparins individually were separately prepared using deionized water or the supplied Reaction Buffer (1 M Tris-HCl, pH 8.0). N-methoxysuccinyl-Ala-Ala-Pro-Val-chloromethyl ketone was used as a control positive inhibitor. The various AAT concentrations and/or heparin formulations (50 µL) were preincubated with 0.4 U/mL pancreatic elastase (100 µL) for 15 minutes. Then 0.1 mg/mL of the substrate DQ^{TM} elastin (50 µL) was then added to the mixture, which upon degradation, releases a fluorogenic signal. After 30 minutes of incubation in the dark, fluorescent intensity was measured immediately using a UV filter (excitation 485 nm and emission 538 nm) and then every 15 minutes for a total of 90 minutes at 37°C in a SpectraMax M2e Microplate Reader and reported as arbitrary fluorescent units (AFU).

Supplemental Figures Legend

Figure S1. Effects of AAT, UFH, or both on elastase activity. **(A)** Inhibition of elastase activity with physiologic concentrations of AAT or with N-methoxysuccinyl-Ala-Ala-Pro-Val-chloromethyl ketone (“Inhibitor”). **(B)** Expanded version of the conditions shown in (A). **(C)** Effects of various concentrations of UFH alone or with AAT on elastase activity. **(D)** Expanded version of the conditions shown in (C). AAT=alpha-1-antitrypsin; UFH=unfractionated heparin.

Figure S2. Effects of enoxaparin or nadroparin alone, or with AAT on elastase activity. **(A)** Effects of various concentrations of enoxaparin alone or with AAT on elastase activity. **(B)** Expanded version of the conditions shown in (A). **(C)** Effects of various concentrations of nadroparin alone or with AAT on elastase activity. **(D)** Expanded version of the conditions shown in (C). Enox=enoxaparin; Nadro=nadroparin.

Figure S3. **Intermolecular interactions and charge complementarity of the TMPRSS2–AAT complex. (A)** TMPRSS2 and TMPRSS2–AAT electrostatic surfaces (blue=positive, red=negative). AAT is shown as a cartoon in the left image and a molecular surface in the right image. A dotted ellipse indicates positively-charged regions on AAT and TMPRSS2 that are predicted to be energetically unfavourable. **(B)** “Open book” representation showing molecular surfaces of complex interface after rotating AAT by 180°. Hydrogen-bonding interactions between the RCL of AAT and **(C)** TMPRSS2 and **(D)** trypsin (bovine cationic S195A trypsin – human AAT Pittsburgh Michaelis complex (M358R; PDB ID 1OPH)) (1). Black broken lines indicate hydrogen bonds.

Figure S4. **Structural agreement between the RCL of AAT and the inhibitory loop of HAI-2.** Model of the complex between TMPRSS2 and an endogenous TMPRSS2 inhibitor, HAI-2, structurally aligned with the TMPRSS2–AAT complex. TMPRSS2 is shown as a transparent

molecular surface (cyan), AAT (magenta) and HAI-2 (wheat) as cartoons. Inset shows a close-up comparison of the inhibitory loops of HAI-2 (wheat) and AAT–RCL (magenta) that interact with TMPRSS2.

Figure S5. TMPRSS2–AAT heparin docking calculations. (A) Surface heatmap color-coded by heparin binding scoring: red (high), white (medium), black (low). Predicted heparin-binding hotspot is located exclusively at the rim of the TMPRSS2–AAT interface. **(B)** Glycosylation sites in the context of heparin binding sites. TMPRSS2–AAT (magenta) complex cartoon. TMPRSS2 domains are colored separately: LDLR-A, plum; SRCR, green; Peptidase S1 (catalytic) domain, cyan. Disulphides are shown as sticks. The catalytic triad (H296, D345 and S441) are shown as red sticks. Asparagine glycosylation sites on both molecules are shown as space-filling models and are labeled. NAG sugar molecule coupled to glycosylation site N213 in the crystal structure of TMPRSS2 (PDB ID: 7MEQ) is indicated. Carbon atoms of each 4mer heparin are colored differently.

Figure S6. Elastase-AAT Michaelis complex model. AAT is an excellent inhibitor of elastase, consistent with good charge complementarity at the interface. An elastase–AAT complex model was constructed using bovine cationic S195A trypsin – human AAT Pittsburgh Michaelis complex (M358R; PDB ID 1OPH). Porcine pancreatic elastase (PDB ID 4YM9) was structurally aligned to trypsin, and P1 methionine (358) of AAT was mutated back to arginine in *Coot* to generate the wildtype sequence. **(A)** left panel shows cartoon representation of elastase (green)–AAT(magenta) complex, right panel shows molecular surface color-coded according to electrostatic potential (blue=positive, red=negative). **(B)** "Open book" representation showing molecular surfaces of complex interface after rotating AAT by 180°.

References

1. Dementiev A, Simonovic M, Volz K, Gettins PG. Canonical inhibitor-like interactions explain reactivity of alpha1-proteinase inhibitor Pittsburgh and antithrombin with proteinases. *J Biol Chem* 2003; 278: 37881-37887.

B) Immunoblot with anti-His antibody

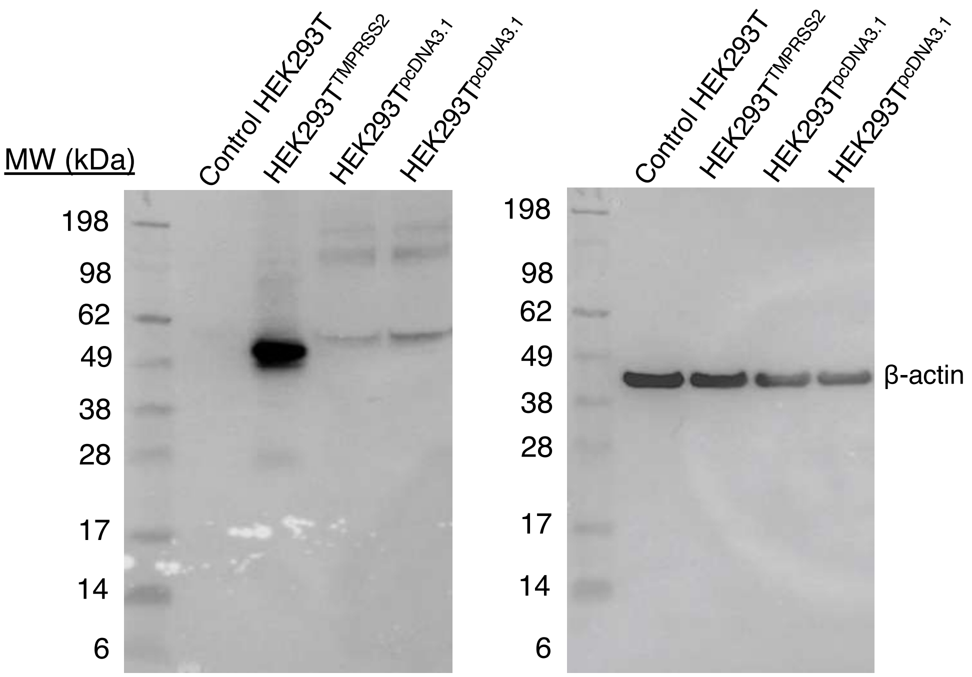

C) Immunoblot with anti-TMPRSS2 antibody

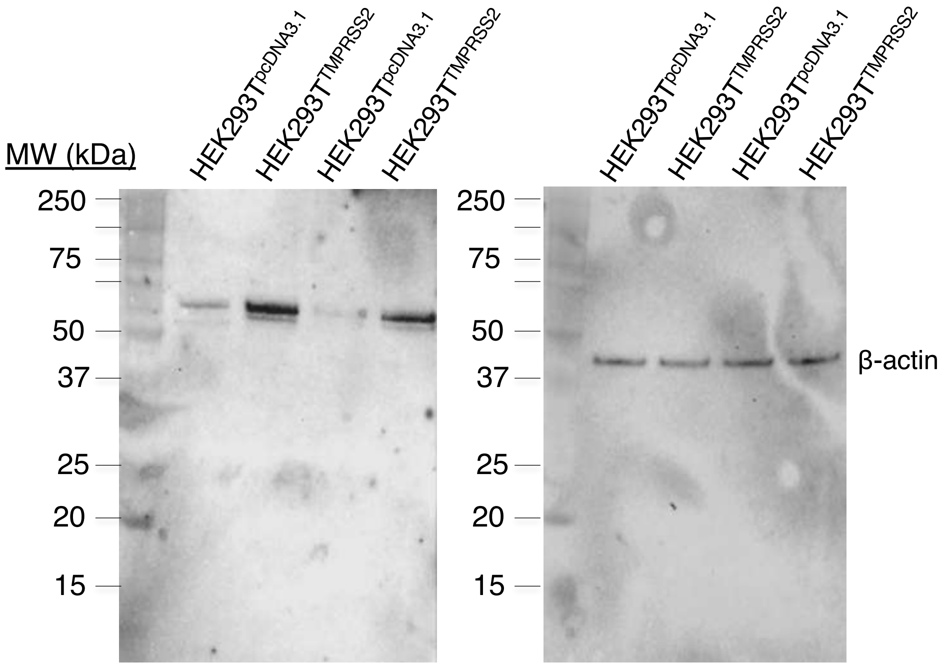

Full Length Western Blots found in Fig 1B and 1C

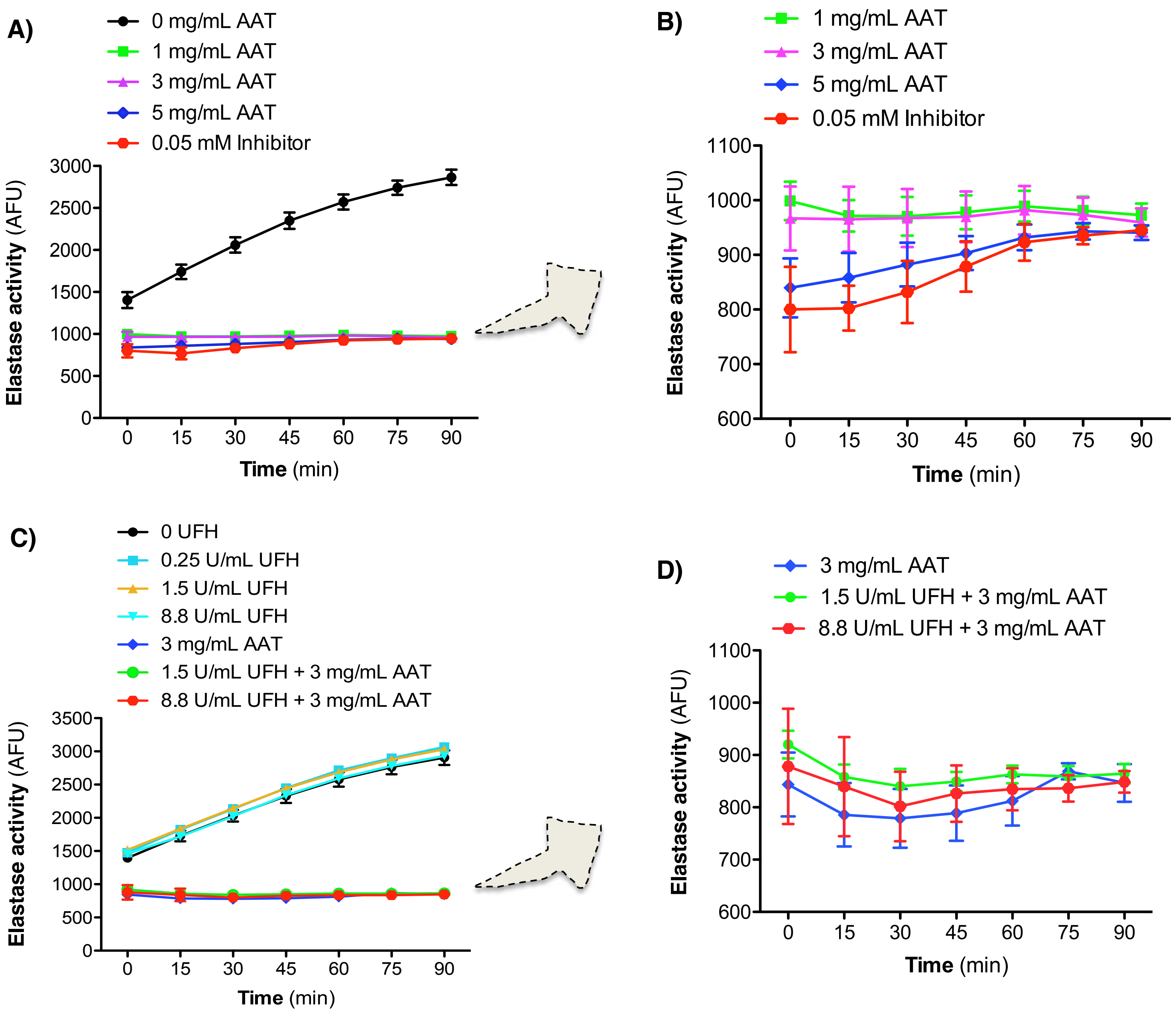

Fig S1
Bai X et al

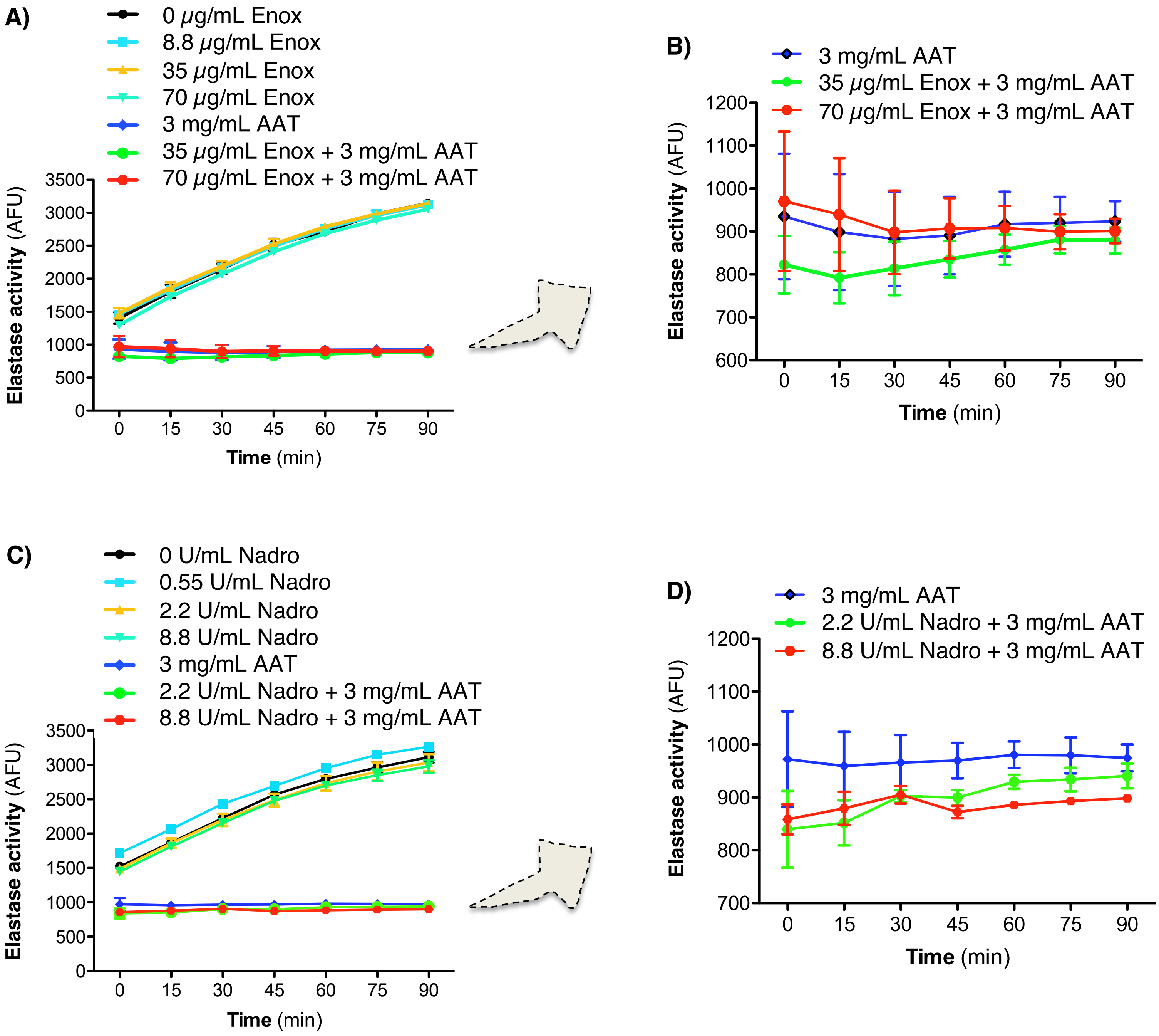

Fig S2
Bai X et al

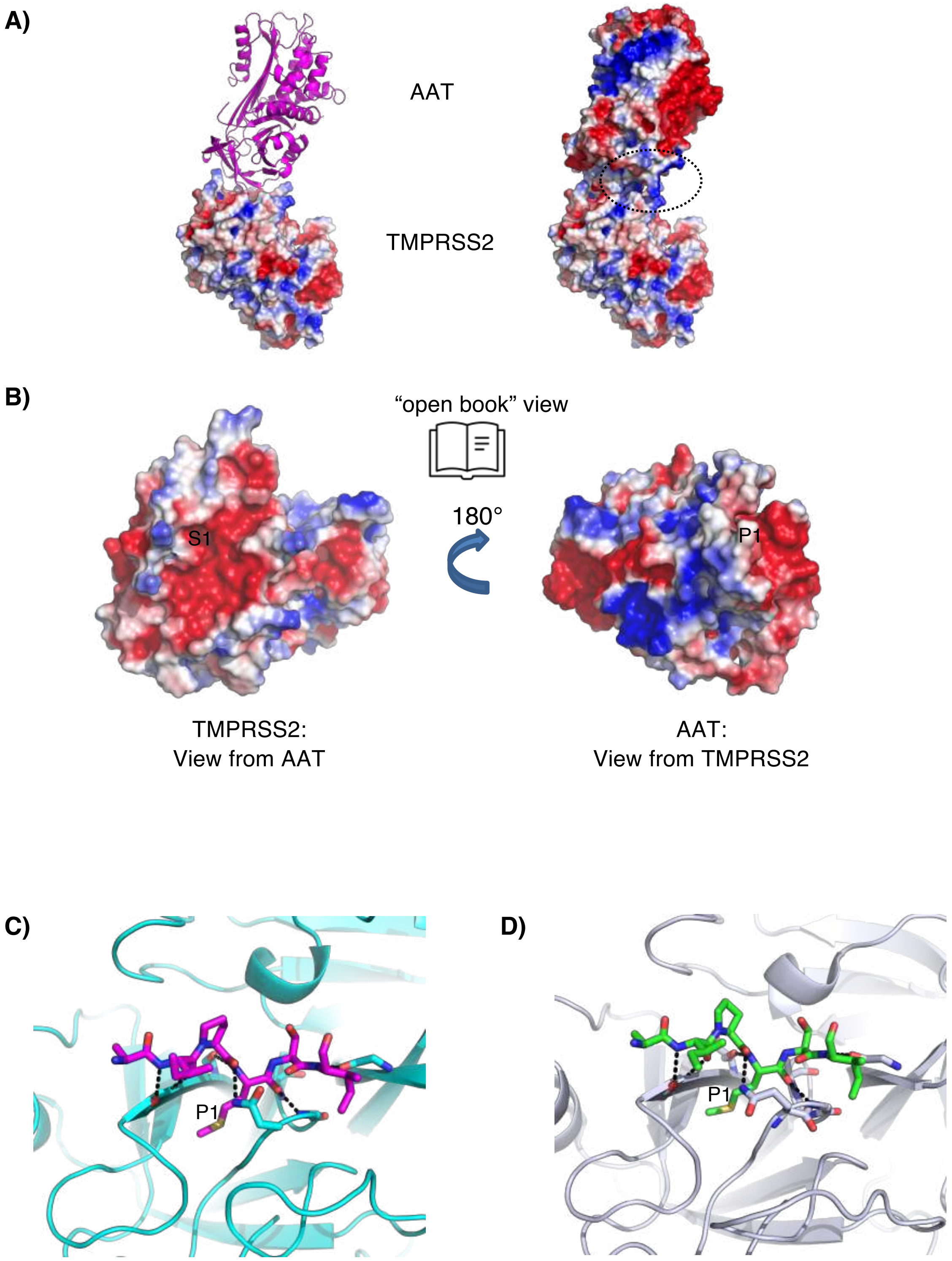

Fig S3
Bai X et al

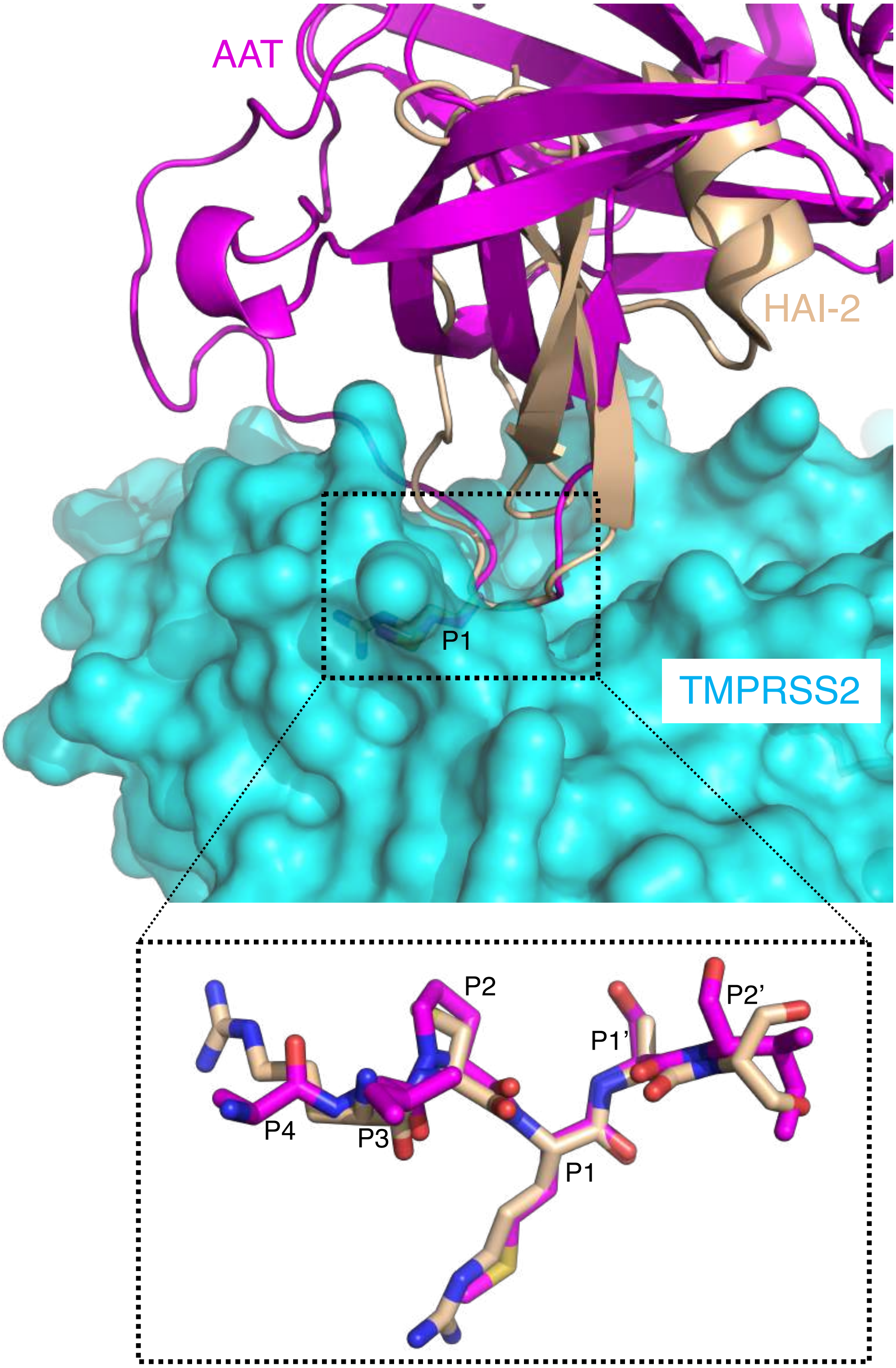

Fig S4
Bai X et al

A)

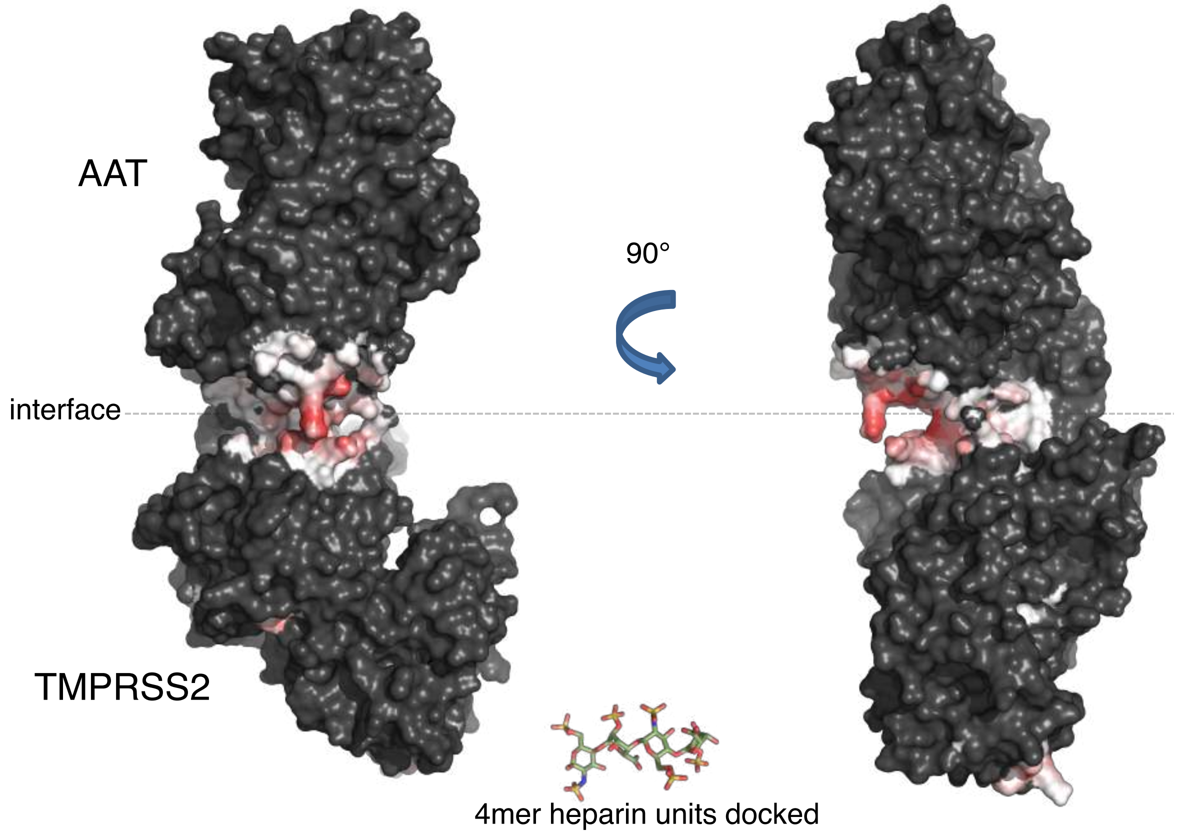

B)

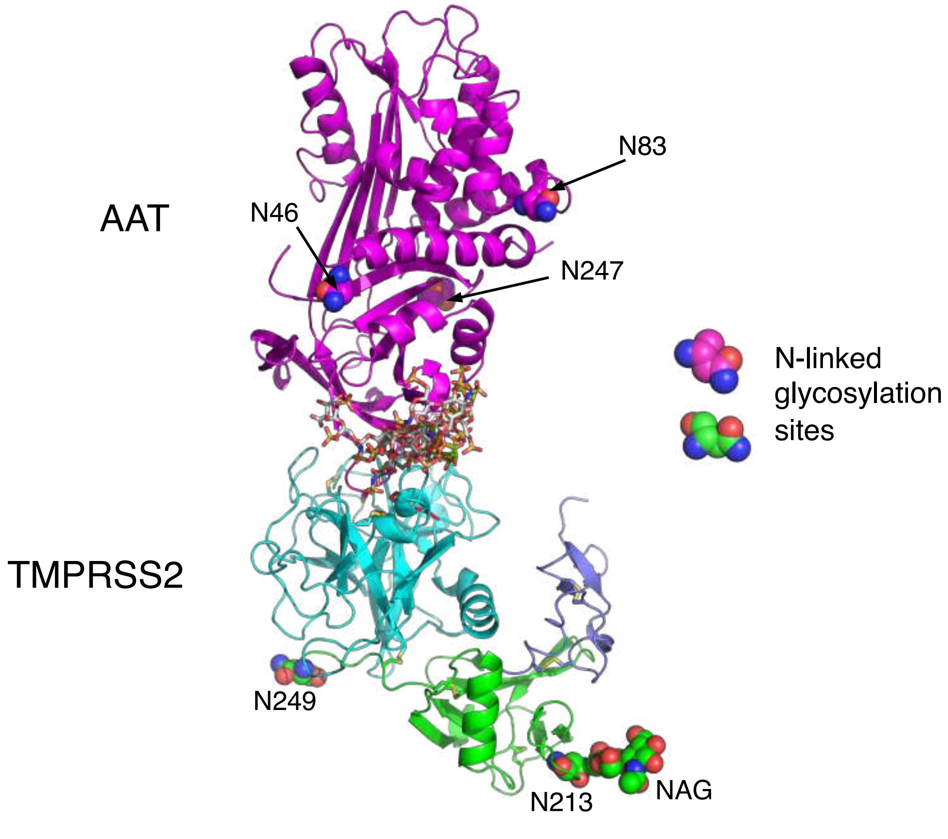

Fig S5
Bai X et al

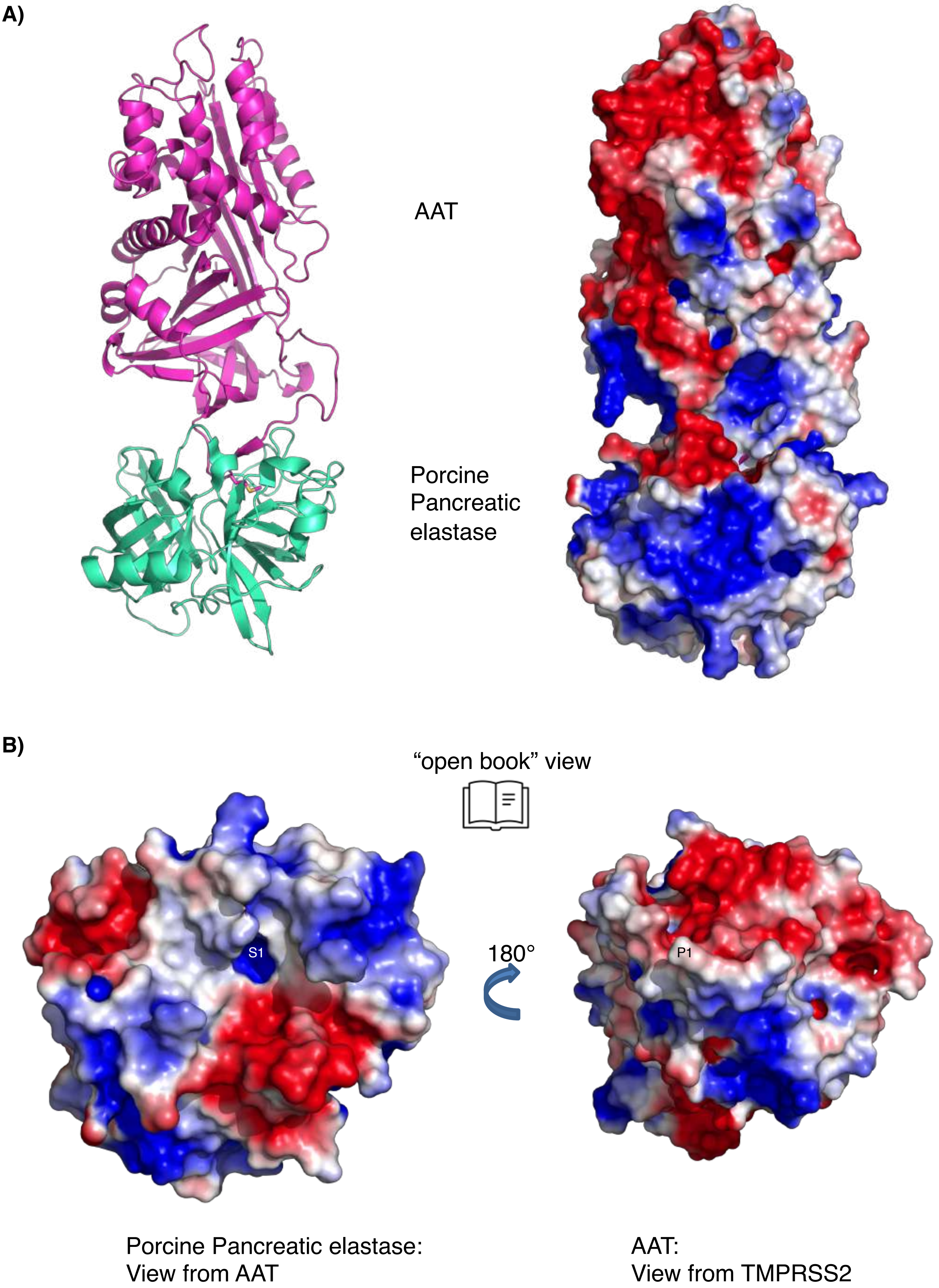

Fig S6
Bai X et al
